# Rare orchid species in Malaysia: New records, recollections and amended descriptions

**DOI:** 10.1371/journal.pone.0267485

**Published:** 2022-04-26

**Authors:** Edward Entalai Besi, Wai Keong Hooi, Runi Sylvester Pungga, Christina Seok Yien Yong, Muskhazli Mustafa, Rusea Go

**Affiliations:** 1 Department of Biology, Faculty of Science, Universiti Putra Malaysia, Serdang, Selangor, Malaysia; 2 2063 Bukit Blossom, Jalan Tok Ungku, Seremban, Negeri Sembilan, Malaysia; 3 International Affairs Division, Kuching, Sarawak, Malaysia; Universiti Teknologi Malaysia, MALAYSIA

## Abstract

*Paphiopedilum exul*, *Calanthe chrysoglossoides*, and *Luisia brachystachys* are reported here as new records for Malaysia, whereas *Bryobium cordiferum* subsp. *borneense*, *Habenaria rostellifera*, and *Taeniophyllum rugulosum* are three rare orchid species recollected from Sarawak, Perlis, and Perak, respectively. This paper highlights brief descriptions and photographic illustrations of each species for easy identification. Besides, notes on morphological comparisons with the closely related species and artificial taxonomic keys are included as well.

## Introduction

Southeast Asia including Peninsular Malaysia and Borneo spans about 3% (4.5 million km^2^) of earth’s total land area that harbours approximately 20–25% of earth’s higher plant species [1, 2]. Substantial anthropogenic habitat alterations, forest fires, and the overexploitation of wildlife in the said vast region are detrimental to its biodiversity [3]. Malaysian vegetation comprises evergreen, montane, mixed deciduous, heath and alpine, limestone, and ultramafic forests supporting a wealth of plant diversity [4]. Worth mentioning is the northernmost corner of Peninsular Malaysia, including Perlis and Kedah, bordered by Thailand in the north constitutes a significant component of the Thai and Burmese flora [5–7]. The forests lie mainly on hilly terrain of limestone, which is part of the Setul and Chuping limestone formation with unique geological formation and high level of flora endemism [8, 9]. However, these forests are declining rapidly because of forest clearance for quarries, timber extraction and other forms of agricultural and infrastructure developments [7, 10–12]. Thus, uncountable number of the plant species may have gone extinct.

Orchidaceae is the most abundant flowering plant family in Malaysia. Approximately, 978 species recorded in Peninsular Malaysia [13–19] and 3,000 species have been recorded in Sabah and Sarawak [13]. Major collective records of Malaysian orchid species were monographed by many botanists, including R. E. Holttum [20], H. N. Ridley [21], J. J. Smith [22], J. J. Vermeulen [23], I. M. Turner [24], G. Seidenfaden, and J. J. Wood [25]. In the past two decades, several studies on the orchid diversity covering various elevation gradients and vegetation types in Malaysia involved local conservationist. Of these, the most significant studies on Malaysian orchids include [12, 14, 26–28]—limestone forest; [13]—peat swamp forest; [29, 30]—hill forest; [31]—lowland forest; [32–36]—montane forest; [37]—coastal heath forest; [38, 39]—logged forest.

Judging by these past and recent works on orchid flora in Malaysia, it is apparent that Malaysia possesses one of the world’s richest orchid floras in one of the largest remaining areas of tropical rainforest in the Old World. Undoubtedly, there are more species still awaiting discovery in the remaining extensive forests of Peninsular Malaysia and Borneo. We are reporting new orchid species to the flora of Malaysia and recollection of additional two rare species aimed at elucidating the diversity of orchids in Malaysia. The rare taxa were found and identified whilst working on the diversity and conservation of wild orchids in the undisturbed and disturbed forests of Malaysia. This paper provides brief description, taxonomic notes, relevant notes on ecology and distribution, morphological comparisons with the closely related species, artificial taxonomic keys, and photographs to facilitate easy identification of these species in the field.

## Materials and methods

Specimen collections were carried with permission from the Forest Department Peninsular Malaysia and Forest Department Sarawak [Access License Ref.—JH/100 Jld. 23 (246); Invitation Letter Ref.—(20)JHS/IAD/600-7/101/Jld.1]. Living specimens were transplanted into an *ex-situ* conservatory, and then further nurtured into identifiable samples. The complete specimens were processed using standard herbarium preparation technique of [40]. Our locality data are withheld to protect the populations from illegal collections. Prior to the morphological examination, methylated spirit-preserved and fresh flower specimens were dissected, described and photographed under AM4113ZT Dino-Lite Digital Microscope. Alpha taxonomy with reference to the type specimens, monographs and protologues was employed in the identification process, evaluation of the species’ distribution status, and a comparative morphology study with the closely similar species. Digitised images of herbarium collections, botanical drawing and records deposited in National Herbarium of the Netherlands (NHN) accessed through Browse Dutch Natural History Collections: BioPortal (Naturalis) (http://bioportal.naturalis.nl/), Herbarium of Singapore Botanic Gardens (SING) accessed through BRAHMS Online managed by University of Oxford (http://herbaria.plants.ox.ac.uk/bol/sing), Swiss Orchid Foundation (https://orchid.unibas.ch/index.php/en/), Kew Herbarium Catalogue (http://apps.kew.org/herbcat/gotoSearchPage.do), Natural History Museum Specimen Collection (https://data.nhm.ac.uk/), Herbarium of Aarhus University (AAU) (https://www.aubot.dk/search_form.php), Museum National D’Histoire Naturelle (MNHN) (https://science.mnhn.fr/all/search), and Plants of the World Online (POWO) (http://www.plantsoftheworldonline.org/) were examined prior to the taxonomic treatment and assessment on range of distribution for each species. The range of distribution from historical and current localities was plotted in Google Earth maps. The accepted names were validated via KEW World Checklist of Selected Plant Families (WCSP) [41].

## Results and discussions

### New records for Malaysia

**Fig 1:**
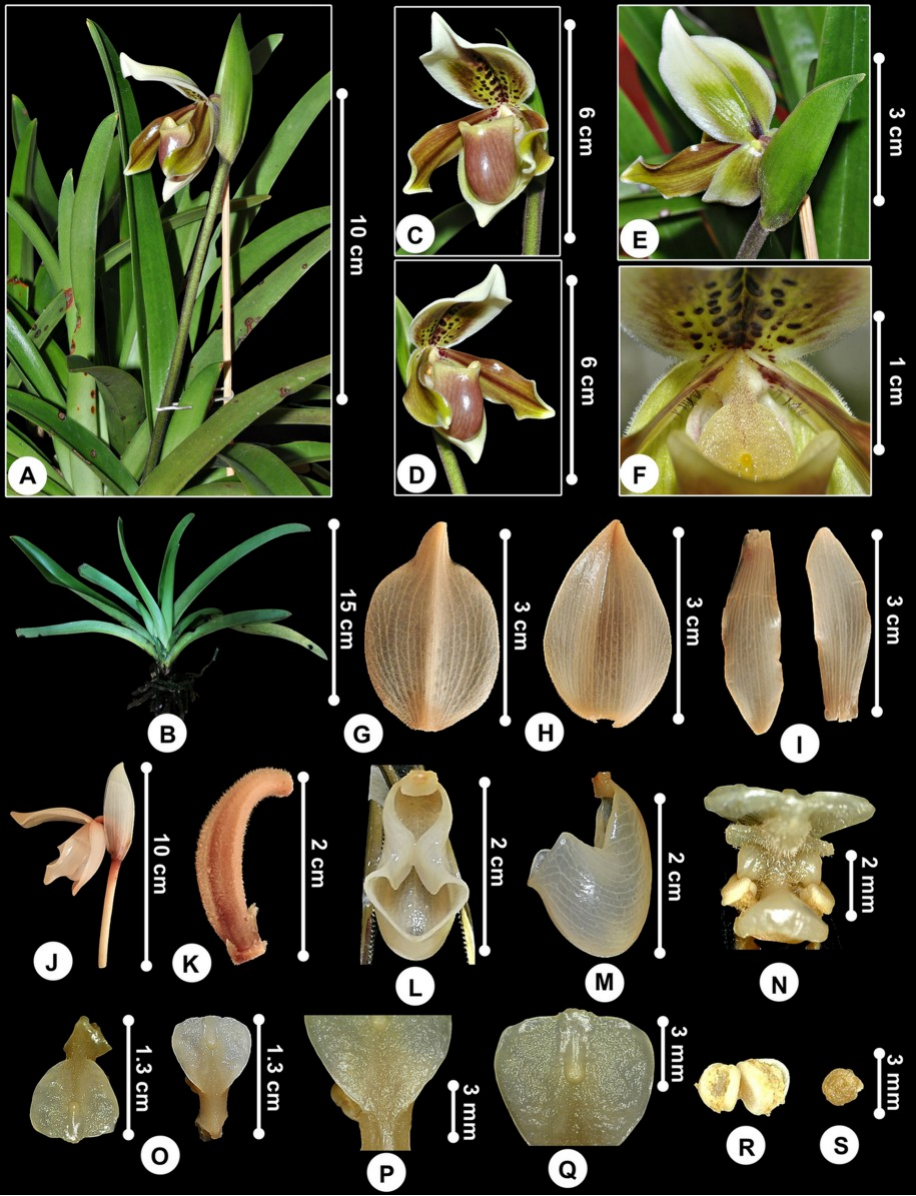
*Paphiopedilum exul*. A. Single flower. B. Plant. C, D, E. Flower from front, lateral and back view. F. Flower front view showing spotted dorsal sepal and hairy staminode. G. Dorsal sepal. H. Dorsal sepal from dorsal and back view. I. Petals. J. Inflorescence. K. Pedicel-with-ovary. L. Labellum from top view. M. Labellum from lateral view. N. Gymnostemium from lateral view showing anthers. O. Staminode. P. Staminode showing base connate to the gymnostemium. Q. Staminode showing mucronulate apex. R. Anthers. S. Pollinarium. Note: G-S are preserved specimens.

***Paphiopedilum exul*** (Ridl.) Rolfe, Orchid Rev. 4: 364 (1896); Fig 1.

Homotypic synonyms:––*Cypripedium insigne* var. *exul* Ridl., Gard. Chron., ser. 3, 10: 94 (1891); *Cypripedium exul* (Ridl.) Rolfe, J. Hort. Cottage Gard., ser. 3, 22: 301 (1892); *Cordula exul* (Ridl.) Rolfe, Orchid Rev. 20: 2 (1912). Type: THAILAND. Central Thailand, Bangkok, 1891, *Ridley SING0056352* (SING-photo!).

Heterotypic synonyms:––*Cypripedium exul* var. *aureum* Rolfe, Orchid Rev. 4: 162 (1896); *Paphiopedilum exul* var. *aureum* (Rolfe) Pfitzer in H.G.A.Engler (ed.), Pflanzenr., IV, 50(12): 75 (1903); *Paphiopedilum exul* f. *aureum* (Rolfe) O.Gruss & Roellke, Orchidee (Hamburg) 51: 420 (2002).

Specimen examined:––MALAYSIA. Perlis, ca. 100 m elev., 31 October 2019, *Besi et al*. *EDW113* (UPM!).

#### Description

*Plants* mostly terrestrial, less often lithophytic, caespitose, ca. 20 cm tall including inflorescence. *Stem* ca. 1 cm, short. *Leaves* 7–10 per shoot, 6–21 × 1.7–2 cm, plain green, green to yellowish green, pale green below, venations faint, strap-shaped to oblong elliptic, apex acute with minute cleft, suberect, rigid, fleshy, canaliculated, conduplicate basally, grooved, glabrous, veins prominent below, margin entire. *Inflorescence* single flowered, ca. 15 cm long, slender, erect, pubescent, green covered with dense dark purple indumentum, pedicel-with-ovary completely covered by a large greenish white bract. *Floral bract* folded, lanceolate, apex acuminate, hairy basally, grooved, veins sunken, greenish white, more than half the pedicel-with-ovary length, 4.9 × 1.4 cm. *Pedicel-with-ovary* ca. 2.4 cm long, curved, pubescent, indumentum white, clavate, ridged, reddish green. *Flower* 4.5 × 3.5 cm, dorsal surface of sepals and petals pubescent with short white and dense indumentum, greenish white in general, sepals suffused green, petals greenish brown with clear dark brown veins arching towards apex, pouch showy and glossy reddish-purple pouch hooded by dorsal sepal. *Dorsal sepal* 3.4 × 2.7 cm, cucullate, broadly ovate; apex cuspidate, folded, recurved, ca. 5.5 mm long; base rounded; ventral side white suffused yellowish brown, glossy dark brown spots; spots suffused over lower half from basal to the central area, arranged in lines, contrast with the yellow background; dorsal side suffused green, blotched dark brown at base, sparsely hairy; prominent, raised median outer keel covered by dark brown hairs; margin white, entire, covered by dense white indumentum. *Synsepalum* 3.5 × 2.1 cm, cucullate, ovate, apex obtuse, base cordate, yellowish green, venation green, bordered white, margin covered by dense white indumentum, back side outwardly keeled, suffused green, blotched dark brown at base as in dorsal sepal. *Petals* 3.4 × 1 cm, more than twice as long as wide, outspread, almost horizontally, oblanceolate, apex obtuse, margins undulate and minutely hairy, proximally black hairs occur on surface at the base, greenish brown at both sides, suffused green towards apex and margins, prominent reddish brown at inner side, minutely spotted at base, recurved forward around the pouch. *Pouch* or *labellum* 2.8 × 1.4 cm, slipper-shaped, side lobes rectangular, incurved, glossy, reddish green, pale green at mouth, venation brown, hairy interior at the nectary and dorsal opening. *Staminode* 8 × 8 mm, obovate, yellowish green, verruculose, hairy, indumentum dark brown, indumentum much longer near the base of the gymnostemium, apex mucronulate, central teeth rounded, base connate to the gymnostemium, anthers and stigma hidden behind staminode, margin slightly recurved; fovea 2 × 0.9 mm, narrowly ovate, yellow, bears a shiny bright yellow knob-like wart at base (umbo); anthers 2, positioned on either one side, 2.5 × 1.4 mm; stigma ca. 4.7 mm, widely ovate. *Pollinia* 2, 2 × 1.3 mm; gymnostemium ca. 5 mm.

#### Distribution

**Fig 2:**
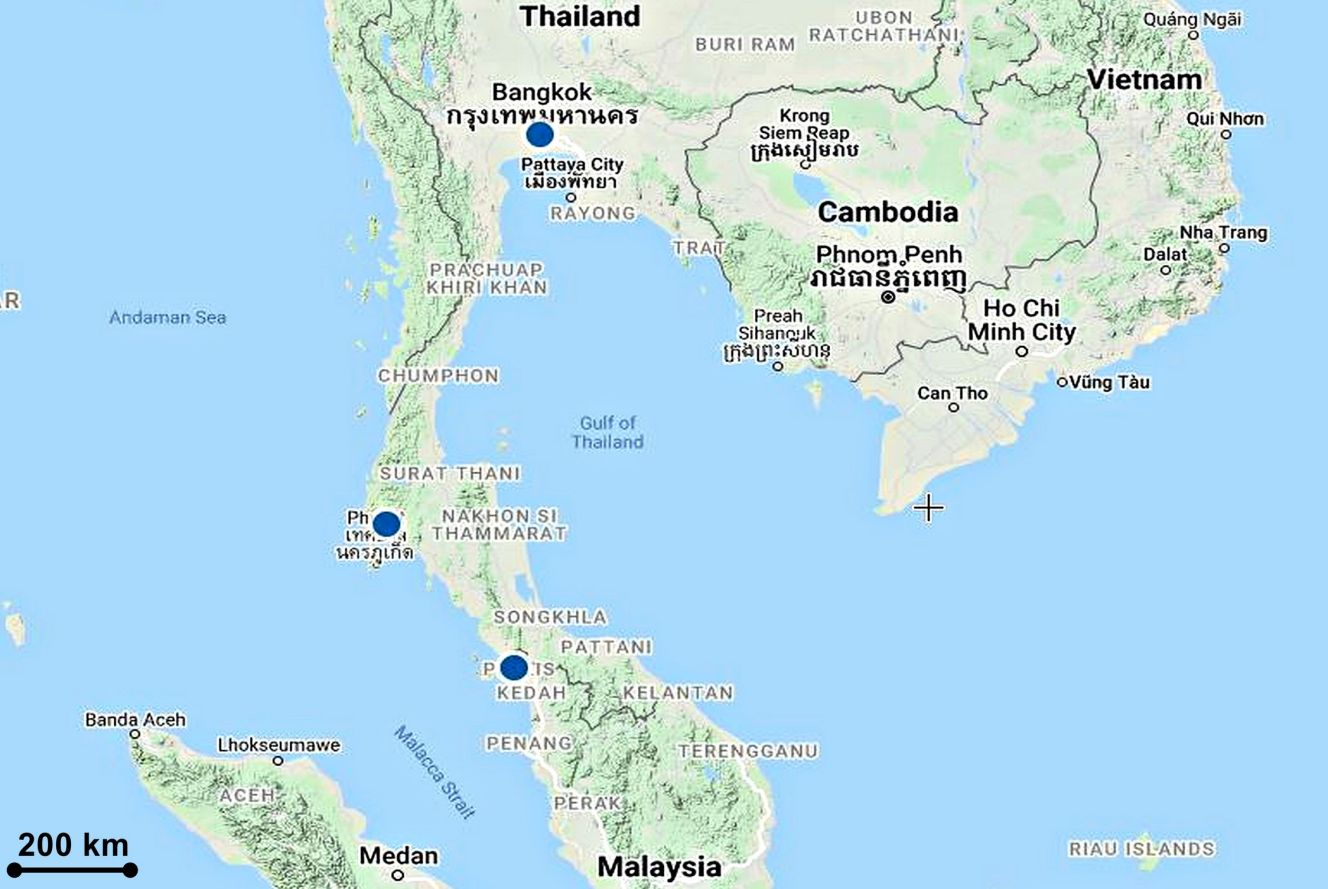
Distribution of *Paphiopedilum exul*.

It was an endemic species to limestone cliffs on the east side of the Phuket-Krabi Gulf of Peninsular Thailand [41, 42]. In Peninsular Malaysia, the specimen of the first record reported in this paper was collected from Perlis (Fig 2). The exact locality is withheld in this paper to protect the population from illegal collections. *P*. *exul* were also reported seen in Kedah, however, we have no authentic specimens to substantiate this claim.

#### Etymology

In Greek, *Paphos* means birthplace of the mythological goddess Aphrodite; and *pedilon* means slipper, referring to the slipper-shaped labellum [43]. The species epithet, *exul* was derived from an English word ‘exile’ by Ridley means ‘banished one’ because of the geographical isolation from the closely allied one, *P*. *insigne* (Wall. ex Lindl.) Pfitzer [43]. Hence, the common name, Excluded Paphiopedilum.

#### Habitat and ecology

**Fig 3:**
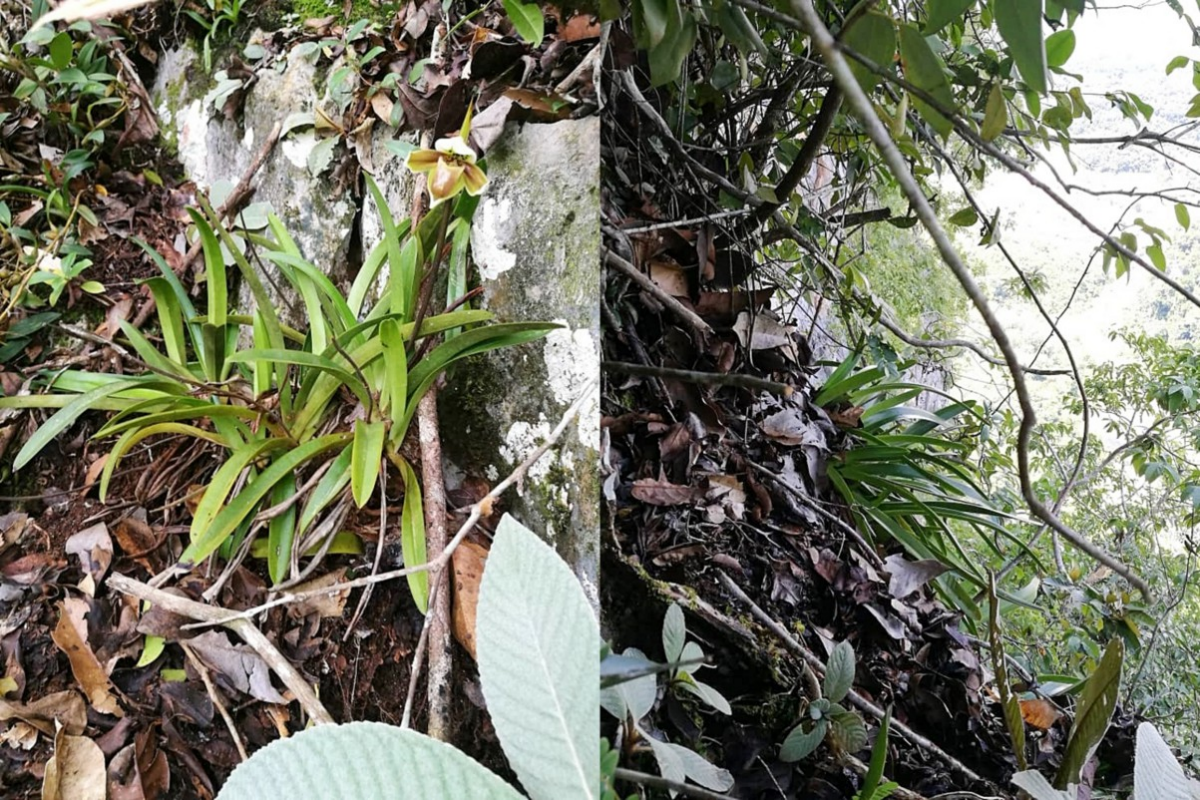
*P*. *exul* growing on the cliff of a limestone forest. Photos by Muhamad Faizal Md Azmi.

It grows on the cliff in a limestone hill forest, dried but shaded (Fig 3).

#### Taxonomic notes

The newly recorded *P*. *exul* belongs to subg. *Paphiopedilum* sect. *Paphiopedilum*. Subg. *Paphiopedilum* sect. *Paphiopedilum* characterised by having single flowered (or at most 2-flowered), linear or spathulate petals that are more than twice as long as broad, pouch side lobes incurved, and strap-shaped and plain green leaves [44]. *Paphiopedilum exul* has been described by several authors under different names due to the wide variation in pouch, staminode and dorsal sepal morphologies. This species closely resembles *P*. *insigne*, but has smaller flowers and belongs in its alliance [45]. The former species differs by having sepals shorter (3.2–3.8 cm vs 5–6.4 cm) [46], staminode apex apiculate instead of emarginated and roundly bilobed, and anthers hidden behind the staminode [47]. In [47], *P*. *exul* differs by having the leaves surface yellowish rather than glaucous as in *P*. *insigne*. However, this character is rather unreliable as leaf colouration changes depending on the habitat and ecological conditions. Plants found in Perlis has slight variations in dorsal sepal and petals and staminode morphologies if compared to the one found in Peninsular Thailand (see Table 1). The base connate to the gymnostemium rather than bilobed and free as illustrated in [47], and the sepals are shorter [46]. Also, the peculiarity seen at the flower size (Table 1). Minor variation in staminode shape, flower size, and leaf pattern across localities are common in *Paphiopedilum* [44, 48, 49]. *Paphioepdilum exul* is having no near allies among the Peninsular Malaysian species. It is easily distinguishable if compared to the other single-flowered with long and spotted petals *Paphiopedilum*, such as *P*. *barbatum* (Lindl.) Pfitzer, *P*. *bullenianum* (Rchb.f.) Pfitzer, and *P*. *callosum* var. *sublaeve* (Rchb.f.) P.J.Cribb.

**Table 1:** Comparison of morphological characters of *P*. *exul* from Perlis and *P*. *exul* from Thailand.

Characters	*P*. *exul* from Perlis (Besi *et al*. EDW113)	*P*. *exul* from Thailand (Besi *et al*. EDW114)	*P*. *exul* from Thailand [46]
Flowers sizes	ca. 3.5 cm across	ca. 5 cm across	ca. 6.5 cm across
Pedicel-with-ovary	pubescent, reddish green, ca. 2.4 cm long	pubescent, reddish green, ca. 4 cm long	pubescent, yellow-green, 2.2–4 cm long
Dorsal sepal shape	broadly ovate, apex cuspidate	broadly ovate, apex cuspidate	ovate-elliptic, apex obtuse
Dorsal sepal size	3.4 × 2.7 cm	3.8 × 2.7 cm	3–4.8 × 2.5–3 cm
Petals shapes	oblanceolate, apex obtuse, margins undulate	oblanceolate, apex obtuse, margins undulate	oblanceolate to narrowly oblong, apex obtuse, margins undulate
Petals sizes	3.4 × 1 cm	4.1 × 1.5 cm	4.3–5 × 1.4–1.7 cm
Synsepalum shape	ovate, apex obtuse	ovate, apex obtuse	oblong-elliptic, apex obtuse
Synsepalum size	3.5 × 2.1 cm	3.8 × 2.3 cm	3.4–4.7 × 1.6–2.5 cm
Pouch colour	glossy, reddish green with brown veins	glossy, reddish green with brown veins	glossy, yellowish-beige with darker veins
Pouch size	2.8 × 1.4 cm	3.5 × 1.5 cm	3–3.5 × 1.9 cm
Staminode shape	obovate, apex mucronulate with the central teeth rounded, base connate to the gymnostemium	obovate, apex mucronulate with the central teeth rounded, base connate to the gymnostemium	obovate, apex obtuse to rarely retuse, base bilobulate rounded and free
Staminode size	8 × 8 mm	8 × 7 mm	6–8 × 7–9 mm

### Artificial key to *Paphiopedilum* subg. *Paphiopedilum* from Peninsular Malaysia, with usually single flowers (or at most 2 flowers) per plant

**Table:** 

Leaves elliptic to oblong-elliptic, yellowish or bluish green, usually mottled; flowers bluish green………………….*P*. *bullenianum*, *P*. *barbatum*, *P*. *callosum* var. *sublaeve* (sect. *Barbata*)
Leaves strap-shaped to oblong-elliptic, plain green; flowers yellowish green…………*P*. *exul* (sect. *Paphiopedilum*)

#### Species reference

*Cypripedium insigne* var. *exul* Ridl. [50]; *Cypripedium exul* var. *aureum* Rolfe [46], *Paphiopedilum exul* var. *aureum* (Rolfe) Pfitzer [47].

#### Additional specimens examined

THAILAND. Peninsular Thailand, Kasoom, Phangnga, Nov 1897, *Curtis SING0141768* (SING-photo!); 1892, *Ridley SING0141766* (SING-photo!); 19 November 1901, Communic. ex. Herb. Hort. Bot. Bog., *L*.*1527086* (NHN-photo!); 19 November 1901, Communic. ex. Herb. Hort. Bot. Bog., *L*.*1527085* (NHN-photo!); 12 December 1918, *Mhd Haniff SING0141767* (SING-photo!); Central Thailand, Bangkok, 2 April 1925, *Kerr K000595625* (K-photo!); 2 August 2020, cultivated *Besi et al*. *EDW114* (UPM!).

**Fig 4:**
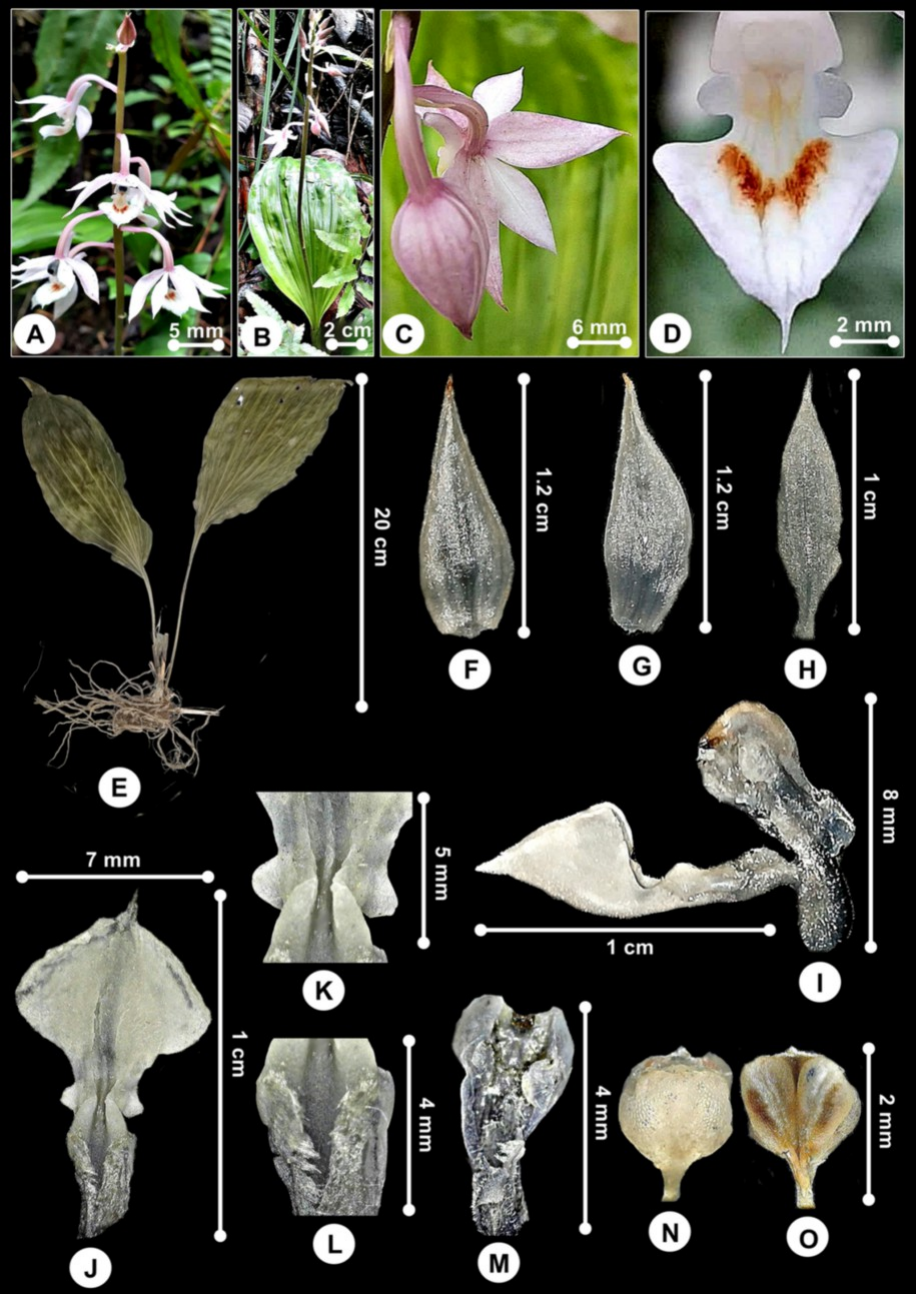
*Calanthe chrysoglossoides*. A. Inflorescence. B. Plant in the natural habitat. C. Dorsal view of the flower. D. Labellum with a central orange mark. E. Plant (dried specimen). F. Dorsal sepal. G. Lateral sepal. H. Petal. I. Lateral view of the labellum and Gymnostemium showing canaliculated claw. J. Labellum (flattened). K. Close-up of the labellum side lobes. L. Pubescent claw. M. Gymnostemium including foot. N. Anther-cap. O. Pollinia. Note: F-O are preserved specimens.

***Calanthe chrysoglossoides*** J.J.Sm., Bull. Dép. Agric. Indes Néerl. 43: 24 (1910); Fig 4.

Type: INDONESIA. Java, Gunung Salabintana (Selabintana), *Jackson 1506*, *1165* (syntype BO-photos!).

Specimen examined:––MALAYSIA. Selangor, Batang Kali, ca. 1000 m elev., 12 October 2020, *Besi et al*. *EDW122* (UPM!).

#### Description

*Plants* terrestrial, ca. 20 tall without inflorescence. *Pseudobulbs* clustered, single-leaved. *Leaves* ovate to wide elliptic, apex acuminate, ca. 20 × 8 cm, abruptly narrowed to a ca. 10–15 cm long and sulcate petiole, plicate, very shortly pubescent beneath. *Inflorescence* ca. 30–40 cm long, arised from pseudobulbs which just forming, emerging together with the leaf; rachis ca. 13 cm long, 10- to 15- flowered placed laxly; floral bracts narrowly triangular, very acute, 5 mm long, greenish yellow, persistent. *Flowers* 2.2 × 1.5 cm, opening widely, white, the sepals flushed purple or pink on the outside, prominent median nerve, labellum pale pink with a central orange mark, glabrous inside, finely pubescent outside, spur short at ca. 3 mm long. *Dorsal sepal* 1.2 × 0.5 cm, oblong-ovate, apex acuminate. *Lateral sepals* 1.2 × 0.4 cm, obliquely elliptic, apex acuminate. *Petals* 1 × 0.3 cm, oblong-ovate, apex acuminate, base cuneate, finely pubescent outside. *Labellum* 1.1 × 0.7 cm, flabellate from a short claw, 3-lobed, side lobes very small and rounded, 1 mm long; midlobe 6 × 7 mm, spade-shaped, almost circular, disk slightly concave, minutely apiculate, base cuneate, a deep median ridge running from the base to apex, a 5 mm long linear and verrucose callus at base; claw short, 6 mm long, canaliculated, wall raised and rounded, sparsely pubescent. *Gymnostemium* 4 mm long including foot, stout, clavate, pubescent below the stigma and on the margins; apex 2.5 × 2 mm; anther-cap 2 × 2 mm, rounded, cucullate; pollinia 2 × 1 mm, triangular, outer surface slightly sulcate.

#### Distribution

**Fig 5:**
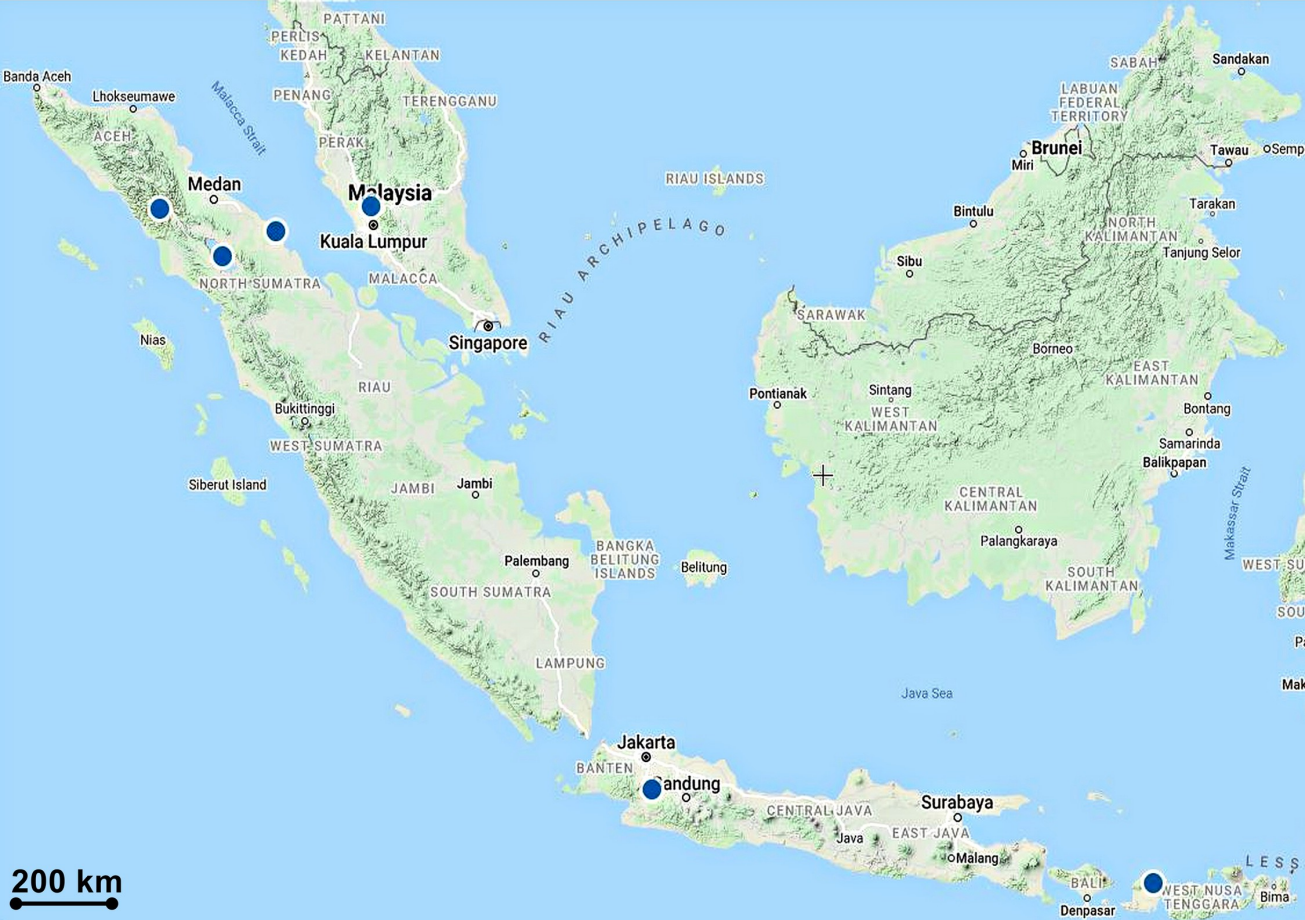
Distribution of *Calanthe chrysoglossoides*.

It was an endemic species to Indonesia, reported from Java, Sumatra, Lesser Sunda Islands, until our recent discovery of a population from Selangor, Malaysia (Fig 5).

#### Etymology

The genus name, *Calanthe* is from the Greek *kalos*, means beautiful, and *anthos*, means flower, in reference to the showy flowers of the type species, whereas the species epithet *chrysoglossoides* is derived from Greek *chrysous*, golden, and *glossa*, tongue, alluding to the colour of the labellum in the type species [43].

#### Habitat and ecology

Growing in a shaded lower montane forest at elevation ca. 1,000 m. An undisturbed forest area but connected to a highly developed area for hotel and shopping complexes.

#### Taxonomic notes

*Calanthe chrysoglossoides* is similar to *C*. *monophylla* Ridl. and *C*. *taenioides* J.J. Sm. that occur in montane forests and belongs to sect. *Monophylla*. The two species were the only representative of the single-leaved *Calanthe* found in Peninsular Malaysia. In comparison, the species are having a creeping rhizome and only one full-sized leaf to a pseudobulb, except *C*. *chrysoglossoides* has larger flowers with broader sepals and petals, and ovate or almost circular labellum midlobe instead of narrow or bilobed at apex.

### Artificial key to *Calanthe* sect. *Monophylla* from Peninsular Malaysia

**Table:** 

1	Flowers glabrous, yellowish white ………………………………………‥*C*. *taenioides*
	Flowers finely pubescent outside, pinkish white………………………………………2
2	Labellum apically bilobed, lobules diverging and rounded……………‥*C*. *monophylla*
	Labellum apically unlobed, apiculate…………………………….*C*. *chrysoglossoides*

#### Species reference

*Calanthe monophylla* Ridl. [51] and *Calanthe taenioides* J.J.Sm. [51, 52].

#### Additional specimens examined

INDONESIA. Sumatra, Toba, Residency of Tapianoeli, Vicinity of Loemban Loboe, 27 July 1936, *Rahmat Si Boeea L*.*1495195* (NHN-photo!); Sumatra, Toba, Residency of Tapianoeli, Headwaters of Aek Mandosi, 29 September 1936, *Rahmat Si Boeea L*.*1495233* (NHN-photo!); Sumatra, Asahan, 3–15 October 1936, *Rahmat Si Boeea L*.*1495231*, *L*.*1495232*, *L*.*1495234* (NHN-photo!); Sumatra, east coast, Aek Boeloe Bolon, 7 November 1936, *Rahmat Si Boeea L*.*1495238* (NHN-photo!); Sumatra, North Sumatra, Gunung Leuser Nature Reserves, Aceh, 22 June 1979, *Wilde et al*. *L*.*1495198* (NHN-photo!); Lesser Sunda Islands, Lombok, 19 June 1909, *Elbert L*.*1495201* (NHN-photo!).

**Fig 6:**
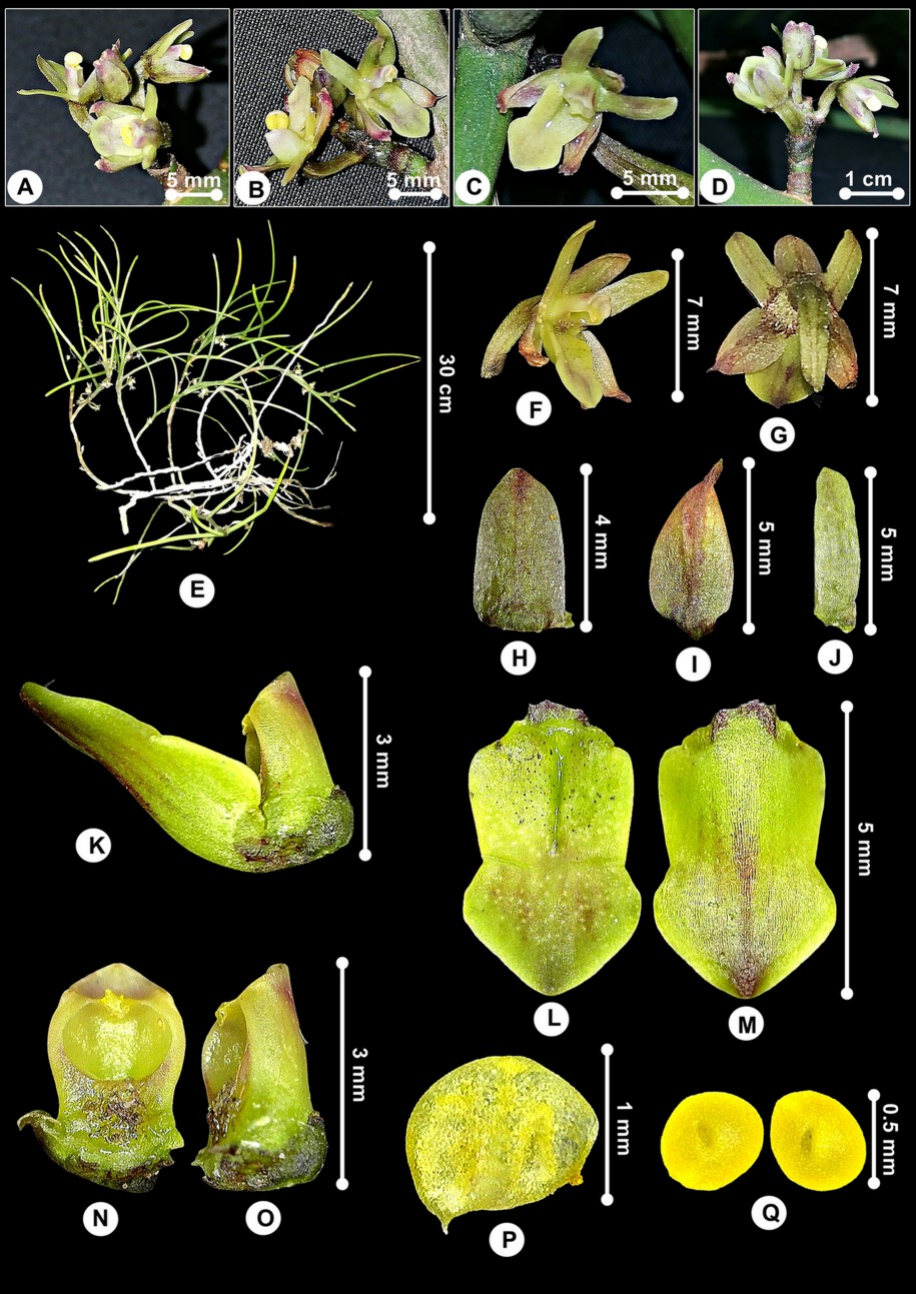
*Luisia brachystachys*. A, B. Flowers from top and lateral views. C. Flower’s front view. D. Inflorescence. E. Plant. F. Flower’s lateral view. G. Flower’s dorsal view. H. Dorsal sepal. I. Lateral sepal. J. Petal. K. Labellum and Gymnostemium. L. Labellum’s abaxial view. M. Labellum’s adaxial view. N. Gymnostemium’s front view. O. Gymnostemium’s lateral view, P. Anther-cap, Q. Pollinia.

***Luisia brachystachys*** (Lindl.) Blume, Rumphia 4: 50 (1849); Fig 6.

Homotypic Names:––*Mesoclastes brachystachys* Lindl., Gen. Sp. Orchid. Pl.: 44 (1830). Type: BANGLADESH. *Wallich K000891542* (syntype K-photo!).

Heterotypic Synonyms:––*Luisia indivisa* King & Pantl., Ann. Roy. Bot. Gard. (Calcutta) 8: 201 (1898); *Luisia siamensis* Rolfe ex Downie, Bull. Misc. Inform. Kew 1925: 384 (1925). Type: THAILAND. Me Sue, near Chiengmai (Chiangmai), 7 April 1912, *Kerr K000891534* (unknown type K-photo!).

Specimen examined:––MALAYSIA. Kedah, Bukit Pedu, ca. 500 m elev., 12 August 2020, *Besi et al*. *HS113* (UPM!).

#### Description

*Plants* epiphytic. *Stems* suberect or curved, branching, lower part brownish grey or glaucous, upper part, greenish, terete, 10–30 cm, stout, ca. 4 mm in diam., internodes 1–2 cm. *Leaves* 5–13 cm × 2–3 mm, green, sometimes glaucous, terete, apex obtuse, straight to slightly recurved at apex. *Inflorescences* 3 to 5 per stem, 2 cm long, 2- to 4-flowered; rachis 1 cm; peduncle 8 mm long, wrinkled, swollen; floral bracts ovate-triangular, ca. 1 mm, fleshy. *Pedicel-with-ovary* 6 mm long, sulcate, greenish brown. *Flowers* opening widely, 7 × 5 mm, sepals and petals yellowish green, abaxial midvein purplish brown, labellum pale yellow with purplish tint at epichilium, Gymnostemium green, purple margined, anther cap yellow spotted with purple; pedicel and ovary pale yellow, tinged with purplish brown, ca. 6 mm. *Dorsal sepal* 4 × 2 mm, ovate, apex obtuse. *Lateral sepals* 5 × 2.5 mm, ovate-oblong, slightly hooded, dorsally slightly carinate, keeled abaxially, keel becoming winged at apex, apex obtuse. *Petals* 5 × 1 mm, linear-oblong, apex obtuse. *Labellum* 5 × 3 mm, glabrous, fleshy, hypochilium concave, deep indentation between epichilium and hypochilium, more or less rectangular, ca. 2.8 × 2.8 mm, indistinct lateral lobes at base; epichilium ca. 2 × 3 mm, suborbicular or heart-shaped, 2 × 3 mm, apex obtuse; hypochilium thick, grooved at the middle. *Gymnostemium* 3 mm long, 2 mm wide at apex, stout; stigma large, orbicular; anther-cap 1 × 2 mm; pollinia 0.5 × 0.5 mm, orbicular.

#### Distribution

**Fig 7:**
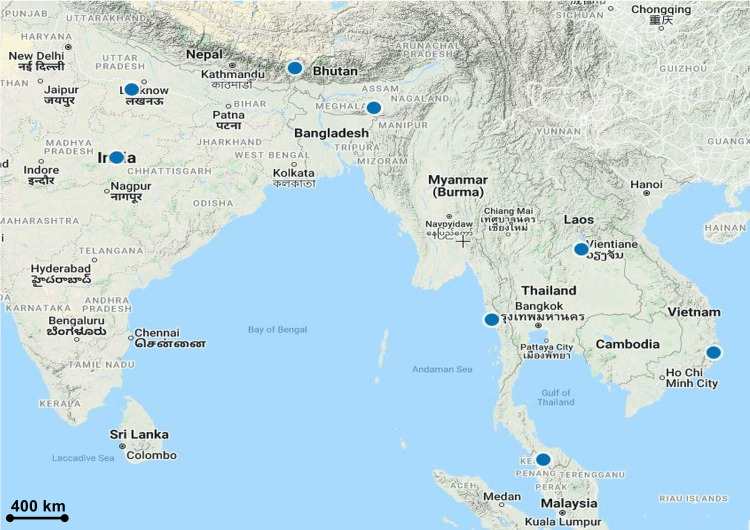
Distribution of *Luisia brachystachys*.

Distributed in Bangladesh, India, Thailand, Myanmar, Vietnam, Laos, Indonesia, and Malaysia. In Malaysia, the plant was collected from Kedah in the northern part of Peninsular Malaysia (Fig 7).

#### Etymology

The genus name commemorates the Spanish explorer Don Luis de Torres (–1493) who was an interpreter for Christopher Columbus on his first voyage to the Caribbean [53]. The species epithet, *brachystachys* is from the Greek, *brachys* means short, whereas *stachys* means an ear of corn [43]; alluding to the short flower spike of this species.

#### Habitat and ecology

Grow on tree trunks at tops of a conglomerate hill forest with elevation of 600 m.

#### Taxonomy notes

*Luisia brachystacys* resembles *L*. *zollingeri* Rchb.f. *in* W.G.Walpers in plant size and having semi-orbicular to heart-shaped epichilium, but differ in having hypochilium separated from the epichilium by a rather indistinct shallow ridge, indistinct and not incurved lateral lobes at base, epichilium lacking of two lateral cushions and upcurved, and narrower oblong (not ovate) petals. On the basis of these morphological variations, the two species can be easily recognized as distinct species if compared to the other *Luisia* species found in Peninsular Malaysia, for examples, *L*. *antennifera* Blume, *L*. *curtisii* Seidenf., *L*. *jonesii* J.J.Sm. Moreover, hypochilium with 5 to 15 nerves and thickly cushioned epichilium are diagnostic characters for *L*. *zollingeri* [54]. Also, the plant that we discovered is very similar to specimens of *De Silva K000873795* (K-photo!) and *Lobb K000873796* (K-photo!), especially on the morphology of the flowers based on the illustrations on the herbarium sheets.

### Artificial key to *Luisia* from Peninsular Malaysia with small flowers ca. 5 mm wide and petals shorter or same length as sepals

**Table:** 

1	Plants and flowers usually large; plants pendant more than 30 cm long; flowers more than 1 cm wide; petals filiform, longer than sepals…………*L*. *antennifera*, *L*. *curtisii*, *L*. *jonesii*
	Plants and flowers small; plants less than 30 cm long; flowers ca. 5 mm wide; petals shorter or same length as sepals…………………………………………………………2
2	Petals ovate, as long as dorsal sepal, together forming a hood; labellum bilobed………………………………………………………………………*L*. *zollingeri*
	Petals linear, narrower than dorsal sepal, 1 mm wide; labellum simple……………*L*. *brachystachys*

#### Species reference

*Luisia zollingeri* Rchb.f. *in* W.G.Walpers [53].

#### Additional specimens examined

THAILAND. Me Sue, near Chiengmai (Chiang Mai), 07 April 1912, 457 m elev., *Kerr K000891534* (K-photo!); INDIA. Sikkim, Dooars, Mount Anduson, March 1897, 457 m elev., *s*. *coll*. *K000891541* (K-photo!); Assam, 01 April 1899–30 April 1899, *Prain’s Collector L*.*1521919* (NHN-photo!); Unknown locality, 1994, *De Silva K000873795* (K-photo!); *Lobb K000873796* (K-photo!); BANGLADESH. *Wallich K000891542* (K-photo!); MYANMAR. Tanintharyi, Dawei, May 1897, *Batten BM000538804* (NHM-photo!); VIETNAM. Anam, Nha Trang, 22 March 1960, *Sigaldi P00324094* (MNHN-photo!); LAOS. Brikhane, Wiengchan (Vientiane), 27 March 1932, *Kerr P00324095* (MNHN-photo!).

### Recollections and amended descriptions

**Fig 8:**
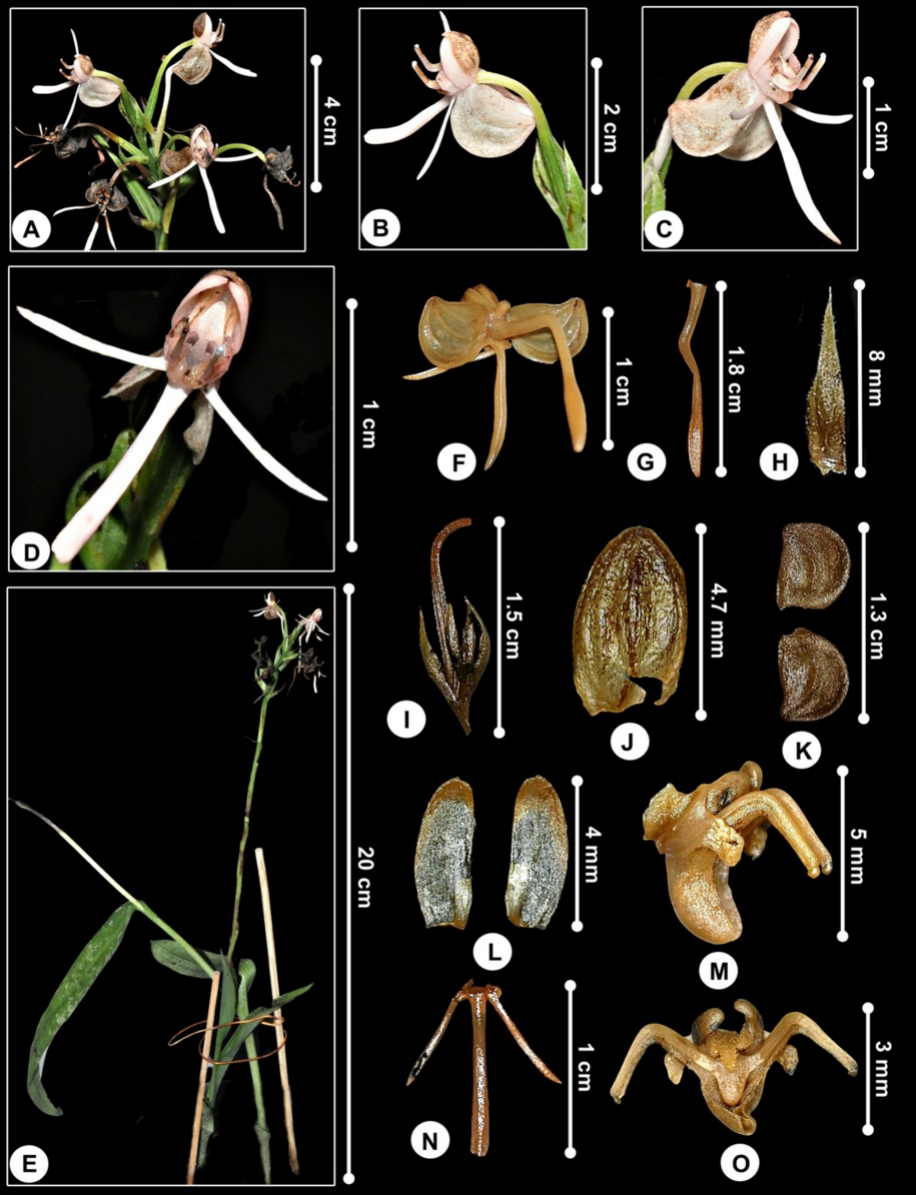
*Habenaria rostellifera*. A. Inflorescence. B, C, D. Flower from lateral and front view. E. Plant. F. Flowers from back view showing spur. G. Spur. H. Floral bract. I. Pedicel-with-ovary and floral bracts. J. Dorsal sepal. K. Lateral sepals. L. Petals. M. Gymnostemium from lateral view showing anther canals. N. Trilobed labellum. O. Gymnostemium from top view showing protruding rostellum. Note: F-O are preserved specimens.

***Habenaria rostellifera*** Rchb.f., Otia Bot. Hamburg.: 34 (1878); Fig 8.

Homotypic synonym:––*Pecteilis rostellifera* (Rchb.f.) M.A.Clem. & D.L.Jones, Austral. Orchid Rev. 83(6): 53 (2018). Type: THAILAND. Peninsular Thailand, Trang, 30 May 1919, *Mhd Haniff SING0047370* (holotype SING-photo!).

Heterotypic synonyms:––*Habenaria hancockii* Rolfe, Bull. Misc. Inform. Kew 1896: 202 (1896); *Habenaria roseata* Ridl., J. Straits Branch Roy. Asiat. Soc. 49: 42 (1908). Type: THAILAND. Peninsular Thailand, Trang, Cultivated in the Penang Gardens, 29 June 1906, *s*.*coll*. *SING0180012* (SING-photo!).

Specimen examined:––MALAYSIA. Perlis, ca. 100 m elev., 2 August 2020, *Besi et al*. *EDW108* (UPM!).

#### Description

Terrestrial. *Plants* up to 20 cm tall. *Stems* ca. 20 cm tall, erect, terete, glabrous, with 2–3 leaves and 1–2 bract-like leaflets. *Cataphylls* up to 2 cm long, tubular, erect, clasping on the stem, with a protruding ovate apex up to 5 mm long, pale greenish with a distinct pale white margins. *Leaves* 12–15 × 1–1.4 cm, spreading, widely spaced, narrowly oblanceolate-oblong, apex acute, often apiculate, adaxial green, abaxial glaucous, pale greenish margins, 3-veined. *Bract-like leaves* 7, up to 2.5 cm long, erect, lanceolate, apex acuminate, basal sheathing, margins minutely denticulate to coarsely, shortly glandular-hairy, green to pale green at apex. *Inflorescences* lax, 7-flowered, often flowered terminally; rachis 3 cm long. *Floral bracts* 4–5, lanceolate, acuminate, 8 × 1.5 mm, shorter than the pedicel-with-ovary, margins shortly hairy. *Pedicel-with-ovary* 1.4 cm long, cylindric-fusiform with a long beak ca. 1.5 mm long. *Flowers* 1.4 × 0.6 cm, pale salmon-pink, lateral sepals with large brown blotch in the middle, fragrant. *Dorsal sepal* 4.7 × 2.6 mm, suborbicular, apex cuspidate, margin entire, shortly hairy. *Lateral sepals* 6 × 5 mm, obliquely ovate to rounded, apex obtuse, reflexed, 3 prominent sunken veins, margins minutely denticulate. *Petals* 4 × 1.5 mm, oblong, apex cuspidate, margin denticulate, shortly hairy, forming a hood with the dorsal sepal. *Labellum* ca. 11 mm long, deeply 3-lobed above a short united part; midlobe linear to oblong, apex rounded, canaliculated, 11 × 1 mm; side-lobes divaricate, linear to oblong, apex attenuate, 7 × 0.5 mm; spur cylindric, nearly as long as the ovary to slightly longer, geniculate, thickened and slightly folded apically, ca. 1.7 cm long. *Gymnostemium* 5.1 mm long; foot ca. 3 mm long; anther hooded by dorsal sepal, canals ca. 5 mm long, geniculate at the middle, narrower towards lobed apex; stigmas ca. 2 mm long, freely projecting, rather short; rostellum 3-lobed with arms ca. 1.7 mm long and a middle 3-lobed tongue protruding, postulate, ca. 1.5 mm long; stelidia oblong, apex rounded, geniculate, ca. 1.7 mm long; wings oblong, grooved, ca. 2 mm long. *Pollinia* not seen.

#### Distribution

**Fig 9:**
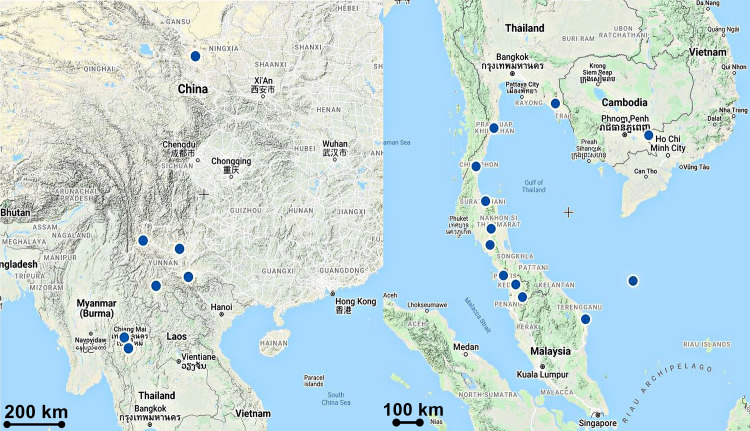
Distribution of *Habenaria rostellifera*.

Cambodia, China South-Central, Laos, Malaya, Thailand, Vietnam [41]. In Peninsular Malaysia, it was found in Perlis (Fig 9).

#### Etymology

In Latin, *Habena* means reins referring to the long, strap-like divisions of the petals and labellum [43]; *rostellifera* derived from rostellum, a beak, referring to the beak-like and protruding rostellum [55].

#### Habitat and ecology

Found in open places in deciduous forest.

#### Taxonomy notes

The *H*. *rostellifera* complexes including *H*. *rostrata* Wall. ex Lindl. and *H*. *erostrata* Tang & F.T. Wang has a labellum with three equal and spreading linear lobes and a transversal structure in front of the spur entrance [55]. Another easily visible diagnostic character is the labellum with its three spreading linear and about equally long lobes and the fairly long anther canals which are geniculate bent upwards. The long and filiform 3-lobed labellum differentiate *H*. *rostellifera* from any other *Habenaria* species found in Peninsular Malaysia, *H*. *acuifera* Wall. ex Lindl., *H*. *carnea* Weathers, *H*. *dentata* (Sw.) Schltr., *H*. *kingii* Hook.f., *H*. *medioflexa* Turrill, *H*. *paradiseoides* J.J.Sm, *H*. *reflexa* Blume, *H*. *rhodocheila* Hance, *H*. *rumphii* (Brongn.) Lindl., and *H*. *singapurensis* Ridl.. Both *H*. *rostellifera* and the following species, *H*. *rostrata*, have ovaries with a prominent beak which can also be clearly seen in the fruiting stage [55]. The tongue in front of the rostellum was regarded as an outgrowth of the labellum as shown by [56]. Reichenbach regarded this protruding structure as part of the rostellum, which the epithet name *rostellifera* was coined after this prominent character [55, 57].

### Artificial key to *Habenaria* from Peninsular Malaysia, has leaves linear and acute, and a labellum with three linear lobes

**Table:** 

1	Leaves wide, more than 2 cm wide, ovate to oblanceolate…………*H*. *acuifera*, *H*. *carnea*, *H*. *dentata*., *H*. *kingii*, *H*. *medioflexa*, *H*. *reflexa*, *H*. *rhodocheila*, *H*. *rumphii*, *H*. *singapurensis*
	Leaves linear to narrowly lanceolate, 0.5 to 1.5 cm wide……………………………….2
2	Flowers white, lateral sepals oblong with acute tips; labellum lobes linear, more than 1 mm wide………………………………………………………………*H*. *paradiseoides*
	Flowers pale salmon pink, lateral sepals ovate to rounded; labellum lobes filiform, 0.5–1 mm wide………………………………………………………………‥*H*. *rostellifera*

#### Species reference

*Habenaria rostellifera* Rchb.f. [57]; *Habenaria roseata* Ridl. [58]; *Habenaria hancockii* Rolfe [59].

#### Additional specimens examined

MALAYSIA. Peninsular Malaysia, Terengganu, Rantau Abang, 1903, *Down SING0139971* (SING-photo!); THAILAND. Siam. Tung Song, 19 July 1929, *Rabil L*.*1516480* (NHN-photo!); Northeast Thailand, Loie, Sitan, 16 August 1948, *Royal Forest Department P00370734* (MNHN-photo!); Chanburi, Makham, 3 June 1965, *Phengklai L*.*1516481* (NHN-photo!); Surat, North of Chumphon, 11 August 1966, 50 m elev., *Larsen*, *Smitinand*, *Warncke L*.*1516479* (NHN-photo!); Unknown locality, 17 August 1984, *Godefroy-Lebeuf K000364319* (K-photo!); Songkla, Haad Yai, Klong Hoy Kong, 21 November 1984, *Maxwell AMES01946805* (AMES-photo!); Chiang Mai, Wieng Haeng, Ban Jong village area, 18 September 1989, 975 m elev., *Maxwell L*.*1516483* (NHN-photo!); Chiang Mai, Muang, Gukao Falls area, 4 October 1989, 525 m elev., *Maxwell L*.*1516482* (NHN-photo!); Trang, 29 June 1906, *Haniff 4300* (SING-photo!); VIETNAM. Rüng Bbon Phu, August 1967, *Dournes P00439735* (MNHN-photo!); CAMBODIA. *Godefroy 14432* (RENZ-photo!); CHINA. Mengtze (Mengzi), Yunnan, June 1983, 1676–1829 m elev., *Hancock K000827011* (K-photo!); Yunnan, Kunming, 17 August 1984, *Bartholomew & Boufford AMES00140709* (AMES-photo!); Yunnan, Yangpi, *Rock AMES00140671* (AMES-photo!); Yunnan, Mengzi, *Henry AMES00140669* (AMES-photo!); June 1983, *Godefroy-Lebeuf K000364319* (K-photo!).

**Fig 10:**
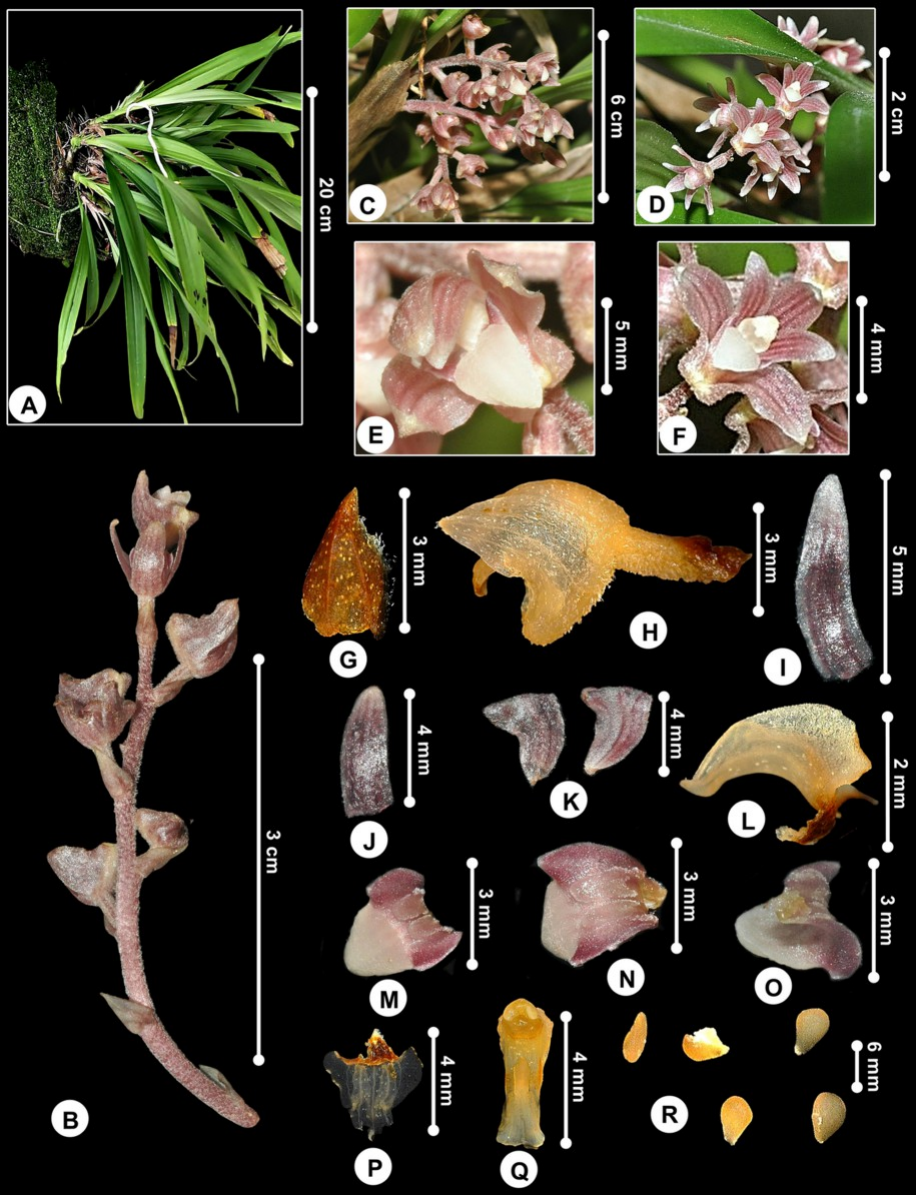
*Bryobium cordiferum* subsp. *borneense*. A. Plant. B, C, D. Inflorescence from lateral and front view. E, F. Flowers showing whitish ovate labellum. G. Floral bract. H. Flower from lateral view. I. Petal. J. Dorsal sepal. K. Lateral sepals. L. Labellum from lateral view. M. Labellum from top view showing midlobe. N. Labellum from top view showing two lateral keels. O. Labellum from top view showing basal part. P. Flattened labellum. Q. Gymnostemium. R. Pollinia.

***Bryobium cordiferum* subsp. *borneense*** (J.J.Wood) Schuit., Y.P.Ng & H.A.Pedersen, Bot. J. Linn. Soc. 186: 193 (2018); Fig 10.

Homotypic synonym:––*Eria cordifera* subsp. *borneensis* J.J.Wood, Kew Bull. 39: 84 (1984). Type: MALAYSIA. Sarawak, Gunung Mulu National Park, Rib Anderson Camp, 14 march 1978, *Nielsen 652* (holotype AAU-Photo!).

Specimen examined:––**MALAYSIA**. Sarawak, Kapit, ca. 50 m elev., 2 December 2019, *Besi et al*. *EDW068* (UPM!).

#### Description

*Plants* epiphytic, up to 20 cm high. *Rhizome* very short. *Pseudobulbs* caespitose, cylindrical, 2-leaved, 2–3 cm long, covered in pale brown, ovate-lanceolate, apex acute to acuminate, membranous sheaths. *Leaves blades* 13–20 × 1.1–1.8 cm, green above, light green below, erect, linear-lanceolate, apex attenuate and unequally bilobulate, conduplicate towards base. *Inflorescence* 6- to 8-flowered, lax, much shorter than leaves; peduncle and rachis pubescent, covered by short white indumentum; peduncle 1.8 cm long, with sterile bract; rachis 3.5 cm long. *Floral bracts* ovate, subacute, minutely puberulus, 3.2 × 1.8 mm. *Pedicel-with-ovary* sessile, terete, densely pubescent, 3.5 mm long. *Flowers* 8 × 8 mm, pubescent dorsally, reddish, veins darker, petals and lateral sepals reflexed. Mentum 3 mm long. *Dorsal sepal* oblong, obtuse, concave, 5.3 × 2 mm. *Lateral sepals* obliquely ovate, falcate, obtuse, cucullate, 5-veined, 4.4 × 3.5 mm. *Petals* linear-oblong, obtuse, falcate, glabrous, 4.6 × 1.2 mm. *Labellum* 3-lobed, strongly recurved, glabrous, 2.6 × 2.8 mm, 4.5 × 3.5 mm when flattened; side lobes erect, widely rounded, ca. 0.9 mm long; midlobe triangular-ovate, acute, fleshy, with widely ovate callus, ca. 1.2 mm long; disc with a median keel, 2 parallel and oblong fleshy lamellae extending from near the base to the base of midlobe, ca. 1.5 mm long; base cuneate. *Gymnostemium* 4.5 × 1.5 mm, clavate, apex 1.5 mm long, foot 3 mm long, dorsal lobe tooth-like, base lobate, stigma 1 × 0.6 mm, anther cucullate, ovate; pollinia 8, ovate, 0.6 × 0.5 mm.

#### Distribution

**Fig 11:**
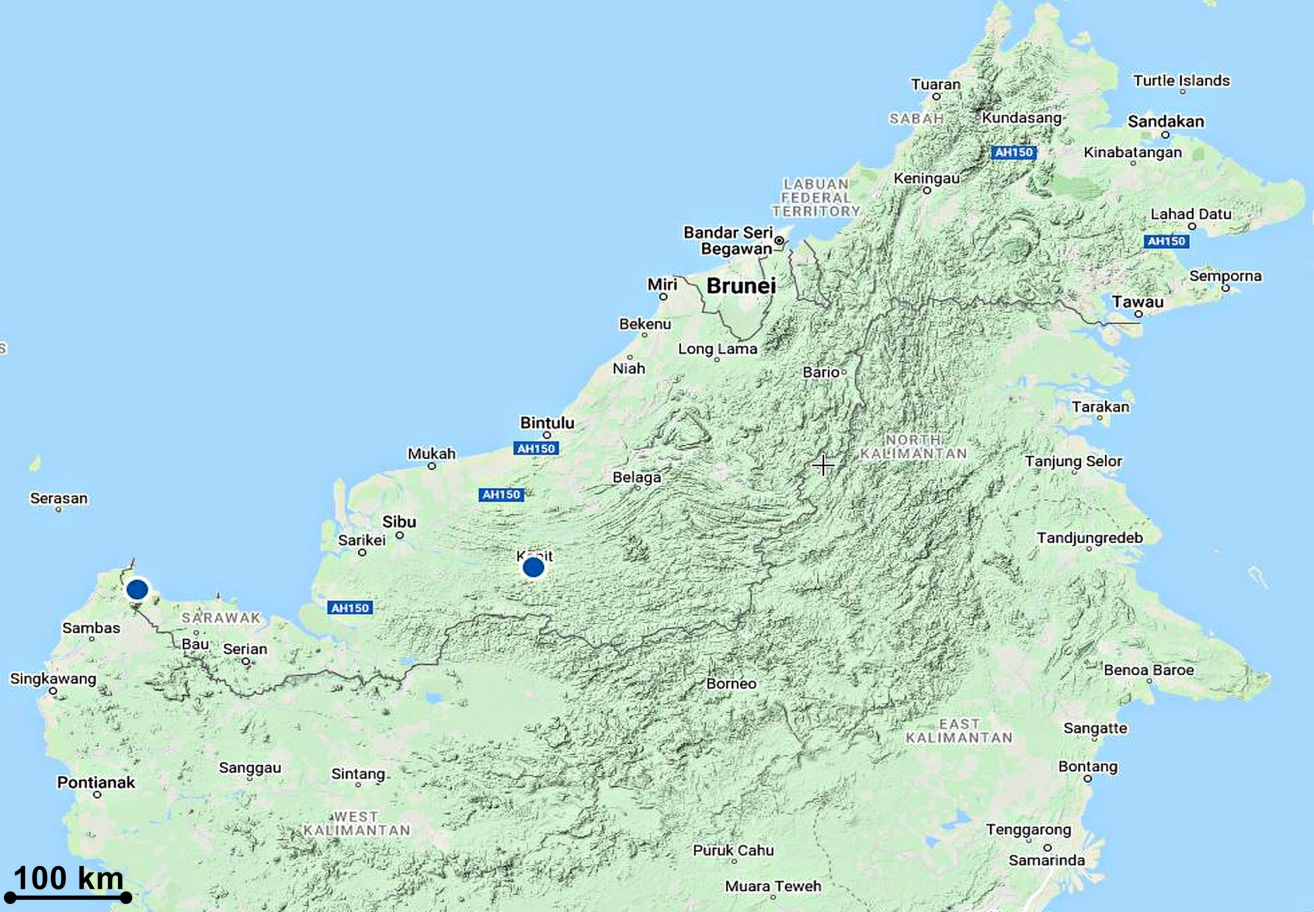
Distribution of *Bryobium cordiferum* subsp. *borneense*.

The first specimen was collected from the 4^th^ Division, Gunung Mulu National Park by Nielsen in 1978 [60]. Since then, there was no further record on its recollection, until now. Our specimen was collected from Kapit, Sarawak (Fig 11).

#### Etymology

In Greek, *bryon* means moss and *bios* as in the English ‘bios’ means life [43]. The species epithet *cordiferum* (latin) means cordate, heart-shaped, with the notch at the base, referring to the heart-shaped labellum [61].

#### Habitat and ecology

**Fig 12:**
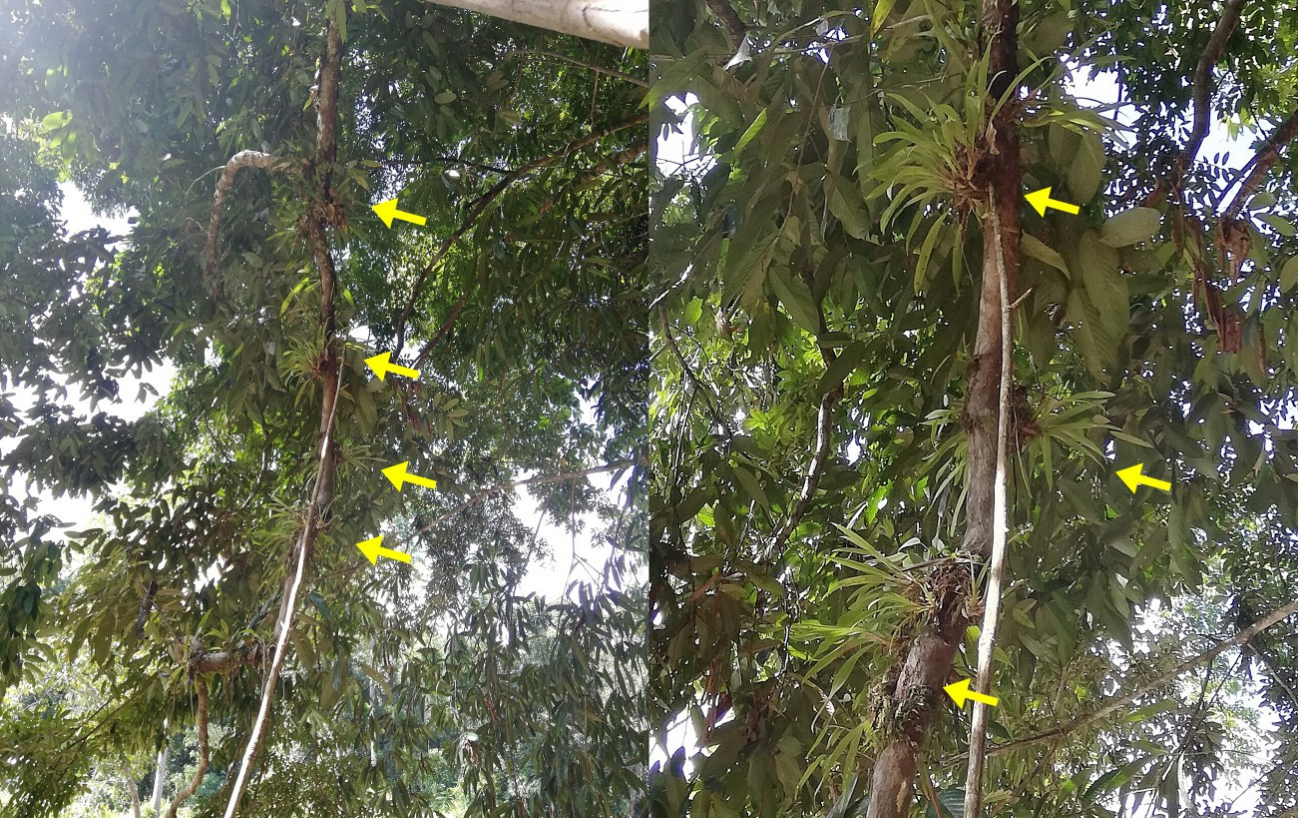
The plants growing as epiphyte on a tree in a riverine forest in Kapit, Sarawak.

Mixed forest on sandy soil with scattered limestone rock [60], and along the riverine forest with alluvial vegetation (Fig 12).

#### Taxonomic notes

According to J. J. Wood in his paper [60], *Bryobium cordiferum* subsp. *borneense* differs from *B*. *cordiferum* subsp. *cordiferum* from Papua New Guinea in having shorter pseudobulbs and leaves, much smaller floral bracts and less hairy flowers [60]. Though our specimen has leaves much wider than Wood’s specimen of *B*. *cordiferum* subsp. *borneense* from Gunung Mulu National Park and Schlechter’s specimen of *B*. *cordiferum* subsp. *cordiferum* (Table 2). But, presumably, both Wood’s and Schlechter’s descriptions were based on dried specimens, hence, the reduced sizes of the specimens. Also, our specimen showing the vegetative structure is very much similar to the subsp. *cordiferum* rather than the subsp. *borneense* (Table 2). However, the bracts and flowers showing affinity to the subsp. *borneense*, especially on the tepals sizes and widely ovate callus on the midlobe (Table 2). *Bryobium cordiferum* differs from the other two species of *Bryobium* species found in Peninsular Malaysia, *B*. *hyacinthoides* (Blume) Y.P.Ng & P.J.Cribb and *B*. *pudicum* (Ridl.) Y.P.Ng & P.J.Cribb, by having pseudobulbs small and inconspicuous, completely covered by rather thick and triangular sheaths.

**Table 2:** Comparison of morphological characters of *B*. *cordiferum* subsp. *borneense* found in Kapit, *B*. *cordiferum* subsp. *borneense* found in Gunung Mulu National Park, and *B*. *cordiferum* subsp. *cordiferum* found in Papua New Guinea.

Characters	*B*. *cordiferum* subsp. *borneense* found in Kapit	*B*. *cordiferum* subsp. *borneense* found in Gunung Mulu National Park [55]	*B*. *cordiferum* subsp. *cordiferum* found in Papua New Guinea [57]
Pseudobulbs size	2–3 cm long	0.8–1.4 cm long	2.5–5 cm long
Leaves shape	linear-lanceolate, apex attenuate	linear-lanceolate, apex acute to attenuate	linear, apex acute
Leaves sizes	13–20 × 1.1–1.8 cm	10–14 × 0.5–0.7 cm	17–25 × 0.5–0.8 cm
Floral bracts shape	ovate, apex subacute	ovate, apex subacute	elliptic, apex obtuse
Floral bracts sizes	3.2 mm long	2.5 mm long	
Dorsal sepal shape	oblong, apex obtuse	oblong, apex obtuse	oblong, apex obtuse
Dorsal sepal size	5.3 mm long	5 mm long	6.5 mm long
Lateral sepals size	4.4 mm long	5 mm long	2.8 mm long
Petals shapes	linear-oblong, apex obtuse	linear-oblong, apex obtuse to acute	obliquely elliptic to strap-shaped, apex obtuse
Labellum size	2.6 × 2.8 mm	4.5 × 3 mm	6.5 × 6.5 mm
Labellum midlobe	widely ovate callus	widely ovate callus	large heart-shaped callus
Labellum lateral lobes	widely rounded	rounded	divergent, triangular, obtuse

### Artificial key to *Bryobium* from Malaysia with caespitose pseudobulbs

**Table:** 

1	Pseudobulbs conspicuous, 6–11 cm long………………………………‥*B*. *hyacinthoides*
	Pseudobulbs caespitose, 1–3 cm long……………………………………………………2
2	Pseudobulbs conical bearing single leaf; flowers scented; labellum midlobe with irregular callus………………………………………………………………‥*B*. *pudicum*
	Pseudobulbs inconspicuous, covered by triangular and alternate sheaths, bearing two leaves; flowers unscented; labellum midlobe with prominent ovate callus…………………………………………………….*B*. *cordiferum* subsp. *borneense*

#### Species reference

*Eria cordifera* Schltr. [62]; *Eria cordifera* subsp. *borneensis* J.J.Wood [60].

#### Additional specimens examined

MALAYSIA. Sarawak, Gunung Mulu National Park, Rib Anderson Camp, 14 march 1978, *Nielsen 652* (holotype AAU-Photo!); PAPUA NEW GUINEA. Kani Gebirges, 10 May 1908, ca. 1000 m elev., *Schlechter L*.*0058744* (isotype NHN-photo!); Kani Gebirges, 10 May 1908, ca. 1000 m elev., *Schlechter K000827429* (isotype K-photo!).

**Fig 13:**
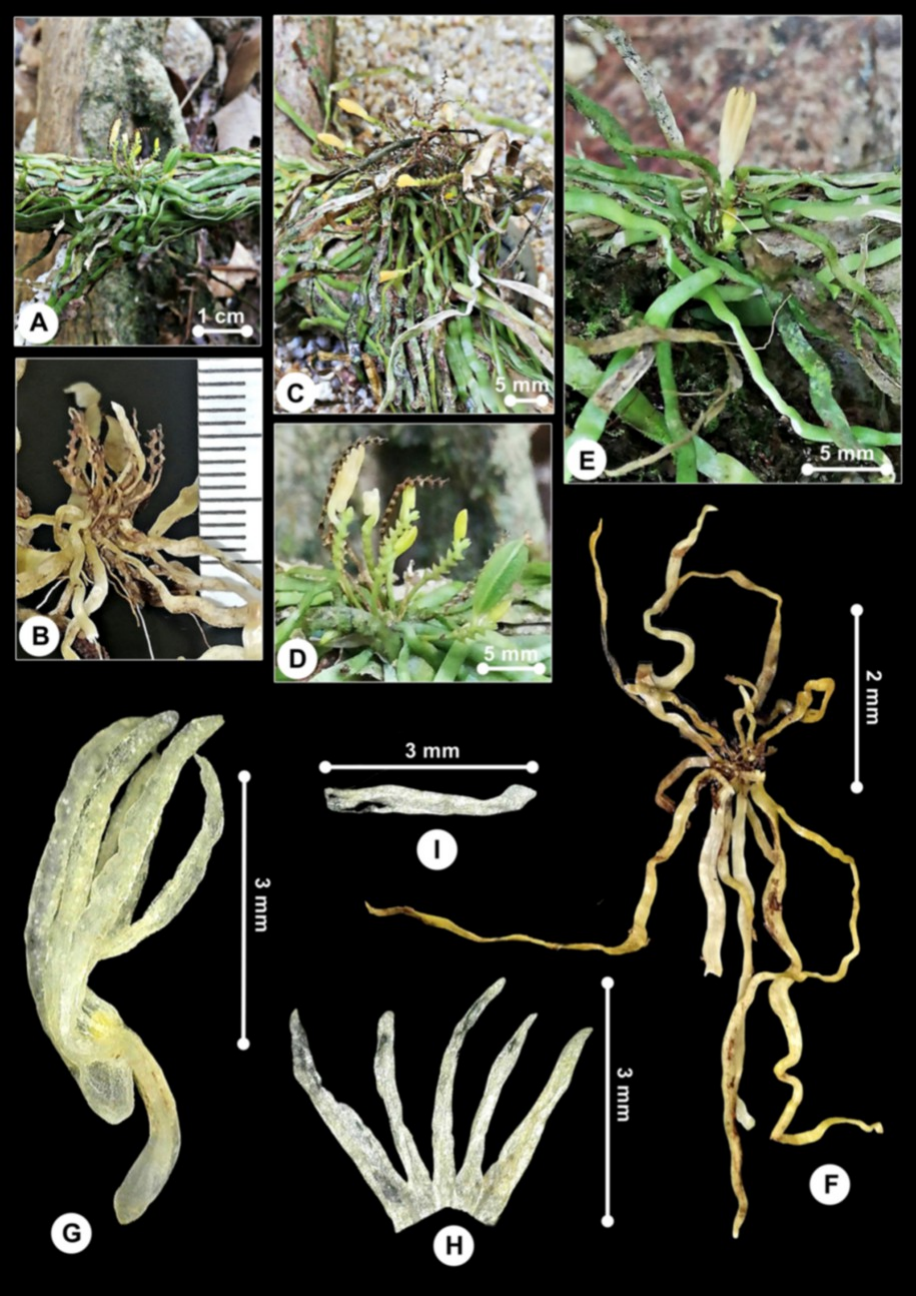
*Taeniophyllum rugulosum*. A. Plant growing on a fallen twig of *Neonauclea* sp. (Rubiaceae) tree in a riverine forest. B, C, D, E. Inflorescence from lateral and front view. F. Plant’s top view showing wrinkled roots (spirit-preserved). G. Flower’s lateral view (spirit-preserved). H. Sepals and petals (spirit-preserved). I. Labellum’s lateral view (spirit-preserved).

***Taeniophyllum rugulosum*** Carr, Gard. Bull. Straits Settlem. 7: 72 (1932); Fig 13.

Type: MALAYSIA. Peninsular Malaysia, Pahang, Sat River, August 1929, *Carr K000942486* (unknown type K-photo!).

Specimen examined:––MALAYSIA. Perak, Lenggong, ca. 100 m elev., 3 April 2021, *Besi et al*. *HS107* (UPM!).

#### Description

*Plants* leafless epiphytic herb forming tangled colonies, often several joined together in a chain, culminates more than 10 inflorescences when flowering. *Roots* thin, flattened, wrinkled, 3–15 × 0.1–0.3 cm, green. *Stems* 1–3 mm long. *Leaves* absent. *Inflorescence* an axillary raceme, ca. 1–1.5 cm long, peduncle filiform, glabrous, apex with alternate and sinuous bracts, green; rachis 5–10 mm long, flattened. *Flowers* 1 or 2 per inflorescence, resupinate, porrect, tubular, 5 × 2 mm, yellowish green, borne successively, spur 1 mm long, tubular; buds, open flowers and capsules often present simultaneously; sepals, petals and labellum almost having the same length, barely distinguishable. *Pedicel-with-ovary* 3 mm long, cylindric. Sepals and petals somewhat fleshy, basally fused, spreading only in upper half, triangular. *Dorsal sepal* free part ca. 3.3 × 0.9 mm, narrowly triangular, apex acute. *Lateral sepals* free part ca. 3.2 × 0.5 mm, triangular, wider at base, apex acute, longer than petals. *Petals* free part ca. 3 × 0.5 mm, narrowly triangular, apex acute. *Labellum* ca. 3 × 0.7 mm, unlobed, pyriform when flattened; apex small, obtuse, upcurved, falcate; spur 1 mm long. *Gynostemium* porrect, at slight angle to ovary, less than 1 mm long. *Capsules* porrect, ellipsoid, ca. 7 mm long.

#### Distribution

**Fig 14:**
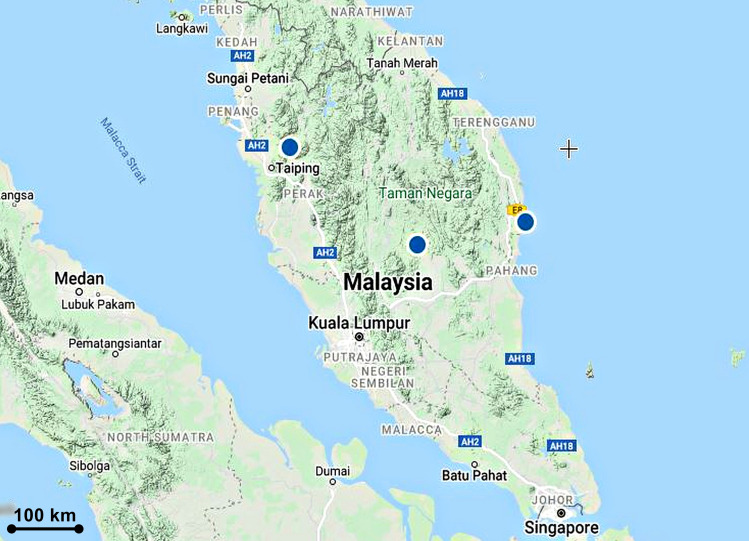
Distribution of *Taeniophyllum rugulosum*.

So far, the species has only been recorded in Peninsular Malaysia and Borneo [25, 41] (Fig 14). However, the occurrence is still not well-known. Due to the inconspicuous appearance of these plants, small, recurrently occurring high in the forest canopy with small short-lived flowers, they are easily overlooked in the field and often preserved in poor conditions [63].

#### Etymology

In Greek, *tainia* means fillet and *phyllon* means leaf, and *Taeniophyllum* referrring to the long filamentous leaves of the plant [43]. Meanwhile, the species epithet, *rugulosum*, in Latin, *ruga* means finely rugose, referring to the roots having many small wrinkles.

#### Habitat and ecology

The plant was growing as epiphytes in a lowland riverine forest, on a common riverine tree. Plants were growing on twigs about 3 cm thick.

#### Taxonomic notes

*Taeniophyllum rugulosum* is belonging to subg. *Codonosepalum* sect. *Sepalocodon*, a group characterised by having sepals and petals adnate at base and bracts alternate [64]. Our specimen is characterised by the flattened roots, flattened 2-ranked and alternate bracts, yellowish-green and flowers 5 mm long, triangular tepals and labellum, sepals and petals basally fused into a tube, and the free part of sepals always longer than tube. Some of these characters are also shared with the closely-related species, *T*. *campanulatum* Carr, *T*. *intermedium* Carr, and *T*. *stella* Carr. The absence of incurved spine on the labellum and the wrinkled roots differentiate *T*. *rugulosum* from *T*. *stella* [63].

### Artificial key to *Taeniophyllum* subg. *Codonosepalum* sect. *Sepalocodon* from Peninsular Malaysia

**Table:** 

1	Rachis short, 3 mm long………………………………………………………………2
	Rachis long, 5–10 mm long……………………………………………………………3
2	Flowers campanulate; free parts of petals and sepals shortly triangular, shorter than the fused part, 1 mm long…………………………………………………*T*.*campanulatum*
	Flowers conical; free parts of petals sepals subulate, adnate at the middle in to a tube, spreading and reflexed, ca. 5 mm long…………………………………………*T*. *stella*
3	Sepals and petals shortly adnate at the base into a tube for a 1 mm long…………………………………………………………………………………………….*T*. *intermedium*
	Sepals and petals adnate at the middle into a tube for a 2.5 mm long……………………………………………………………………………………………….*T*. *rugulosum*

#### Species reference

*Taeniophyllum campanulatum* Carr [64], *Taeniophyllum intermedium* Carr [64], *Taeniophyllum stella* Carr [64].

#### Additional specimens examined

MALAYSIA. Pahang, Tembeling, Sat River, August 1929, ca. 152 m elev., *Carr K000942486* (K-photo!).

## Supporting information

S1 File Comparison on distinctive morphological characters. A. *Paphiopedilum* Subgenus *Paphiopedilum* from Peninsular Malaysia, with usually single flowers (or at most 2 flowers) per plant. B. *Calanthe* Sect. *Monophylla* from Peninsular Malaysia. C. *Luisia* from Peninsular Malaysia with small flowers ca. 5 mm wide and petals shorter or same length as sepals. D. *Habenaria* from Peninsular Malaysia, has leaves linear and acute, and a labellum with three linear lobes. E. *Bryobium* from Malaysia with caespitose pseudobulbs. F. *Taeniophyllum* Subgenus *Codonosepalum* Section *Sepalocodon* from Peninsular Malaysia.
(DOCX)

S2 File List of specimens examined, including information on localities, collectors, date of collection, and habitat. A. *Paphiopedilum exul* (Ridl.) Rolfe. B. *Calanthe chrysoglossoides* J.J.Sm.. C. *Luisia brachystachys* (Lindl.) Blume. D. *Habenaria rostellifera* Rchb.f.. E. *Bryobium cordiferum* subsp. *borneense* (J.J.Wood) Schuit. F. *Taeniophyllum rugulosum* Carr.
(DOCX)
