# Supplementary material for: Rare orchid species in Malaysia: New records, recollections and amended descriptions
Source: PLoS One. 2022 Apr 26;17(4):e0267485. doi: 10.1371/journal.pone.0267485 (PMC9041863; doi:10.1371/journal.pone.0267485)
Supplement: S1 File — A. Paphiopedilum Subgenus Paphiopedilum from Peninsular Malaysia, with usually single flowers (or at most 2 flowers) per plant. B. Calanthe Sect. Monophylla from Peninsular Malaysia. C. Luisia from Peninsular Malaysia with small flowers ca. 5 mm wide and petals shorter or same length as sepals. D. Habenaria from Peninsular Malaysia, has leaves linear and acute, and a labellum with three linear lobes. E. Bryobium from Malaysia with caespitose pseudobulbs. F. Taeniophyllum Subgenus Codonosepalum Section Sepalocodon from Peninsular Malaysia. (DOCX) [file pone.0267485.s001.docx]

**S1 File. Comparison on distinctive morphological characters.** A. *Paphiopedilum* Subgenus *Paphiopedilum* from Peninsular Malaysia, with usually single flowers (or at most 2 flowers) per plant. B. *Calanthe* Sect. *Monophylla* from Peninsular Malaysia. C. *Luisia* from Peninsular Malaysia with small flowers ca. 5 mm wide and petals shorter or same length as sepals. D. *Habenaria* from Peninsular Malaysia, has leaves linear and acute, and a labellum with three linear lobes. E. *Bryobium* from Malaysia with caespitose pseudobulbs. F. *Taeniophyllum* Subgenus *Codonosepalum* Section *Sepalocodon* from Peninsular Malaysia.

**A.**

| Characters | *P. bullenianum*(Rchb.f.) Pfitzer [1] | *P. barbatum*(Lindl.) Pfitzer [1] | *P. callosum*var.*sublaeve*(Rchb.f.) P.J.Cribb [1] | *P. exul* (Besi *et al*. EDW113) |
| --- | --- | --- | --- | --- |
| Leaves number | 3–8 | 3–7 | 3–8 | 7–10 |
| Leaves shape | Narrowly elliptic, elliptic to oblong elliptic, apex acute to obtuse | Narrow to oblong elliptic, apex acute | Narrow to oblong elliptic, apex acute | Strap-shaped to oblong elliptic, apex acute with minute cleft, canaliculated, conduplicate basally |
| Leaves size | 4.7–22.8 × 1.3–3.3 cm | 5–15.5 × 1.6–3.6 cm | 5–15.5 × 1.6–3.6 | 6–21 × 1.7–2 cm |
| Leaves colour | Whitish, yellowish or bluish green above, obscurely to mottled darker green, something without mottling, whitish to grey-green below, often streaked purple | Pale, yellowish or grey green above, obscurely to distinctly mottled dark green, whitish to grey-green below | Pale, yellowish or grey-green above, obscurely to distinctly mottled medium to dark green | Plain green, green to yellowish green, pale green below, venations faint |
| Leaves texture | Glossy or matt, thinly coriaceous to coriaceous, glabrous | Glossy or matt, thinly coriaceous to coriaceous, glabrous | Thinly coriaceous to coriaceous, glabrous | Rigid, fleshy, grooved, glabrous, veins prominent below |
| Leaves margin | Entire | Entire to slightly undulating, at the sparsely ciliate with white indumentum | Basal margin sparsely ciliate, hairs white, the rest of the margin entire | Entire |
| Inflorescence s | 1-(rarely -2) flowered, erect; peduncle 19.5–45.5 cm long, brownish purple, pubescent, indumentum white and purple | 1-(rarely -2) flowered, erect to arcuate; peduncle (20-)30–40(–61.5) cm long, purple to brownish purple, pubescent, indumentum white and purple | 1-(rarely -2) flowered, erect to arcuate; peduncle 13–20 cm long, purple to brownish purple, pubescent, indumentum white and purple | Single flowered, ca. 15 cm long, slender, erect, pubescent, green covered with dense dark purple indumentum, pedicel-with-ovary completely covered by a large greenish white bract |
| Floral bracts | 1.6–3.8 × 0.8–1.2 cm, pale green, suffused purple at the fold and towards base, narrowly ovate, sparsely hairy, margin ciliate, apex acute | 2–3.2 × 1–1.4 cm, pale green, suffused purple at the fold and towards base, narrowly ovate, apex acute, sparsely hairy, margin ciliate | 2–3.2 × 1.4–2 cm, pale green, suffused purple at the fold and towards base, narrowly ovate, apex acute, sparsely hairy, margin ciliate | 4.9 × 1.4 cm, folded, lanceolate, apex acuminate, hairy basally, grooved, veins sunken, greenish white, more than half the pedicel-with-ovary length |
| Flowers sizes | 6.5–9 × 8–9 cm | 7.5–9 × 8–10 cm | 5.9–7.6 × 6.6–8.6 cm | 4.5 × 3.5 cm |
| Dorsal sepal shape | Ovate, concave and hooded; apex acute | Ovate to very broadly ovate, rarely cordate; apex acute to obtuse, sometimes cuspidate | Very broadly ovate to suborbicular; apex acute to obtuse, sometimes cuspidate | Cucullate, broadly ovate; apex cuspidate, folded, recurved |
| Dorsal sepal size | 2.6–4.0 × 1.9–2.5 cm | 3.2–4.4 × 3.5–5.3 cm | 3.5–4.5 × 3.2–4.5 cm | 3.4 × 2.7 cm |
| Dorsal sepal texture | Ventral surface glabrous, dorsal surface sparsely hairy | Ventral surface glabrous, dorsal surface sparsely hairy | Ventral surface glabrous, dorsal surface sparsely hairy | Ventral surface glabrous; dorsal side sparsely hairy; prominent, raised median outer keel covered by dark brown hairs; |
| Dorsal sepal colour | Yellowish to pale green, usually with a lateral band of purple just above the base | White with a middle lateral purple band, often green at the base | White with a middle lateral purple band, often green at the base | Ventral side white suffused yellowish brown, glossy dark brown spots; spots suffused over lower half from basal to the central area, arranged in lines, contrast with the yellow background; dorsal side suffused green, blotched dark brown at base |
| Dorsal sepal margin | Ciliate, basal margin reflexed, apical margin infolded | Ciliate, sometimes slightly undulate or reflexed, occasionally recurved and slightly folded at the apex | Ciliate, slightly undulate or reflexed, occasionally recurved and slightly folded at the apex | Entire, white, covered by dense white indumentum |
| Petals shapes | Spathulate to oblanceolate, slightly twisted on the apical half | Linear oblong, slightly wider near apex, apex acute, base slightly inflexed and/or slightly twisted upwards | Linear oblong, slightly wider near apex, apex acute, base slightly inflexed with upper margin arcuate | Oblanceolate, apex obtuse, recurved forward around the pouch |
| Petals sizes | 3.6–5.2 × 0.9–1.5 cm | 4.5–5.2 × 0.7–1.5 cm | 4.0–5.3 × 1.0–1.3 cm | 3.4 × 1 cm |
| Petals texture | Often with one or two teeth at the distal end near apex | Warts with irregular shapes on upper margins and blade, hairy at margins | Warts with irregular shapes on upper margins, hairy at margins | Proximally black hairs occur on surface at the base |
| Petals margins | Entire, upper margin of basal half sinuate | Ciliate with purple hairs of uneven length; warts blackish purple to black, glossy, usually on upper margins | Ciliate with purple hairs of uneven length and blackish warts | Margins undulate and minutely hairy |
| Petals colour | Pink to purple, with many random and irregular maroon to black spots in basal half | Variable, combination of green, pink, brown and purple, sometimes almost completely purple, basal often spotted black, apex white | Pink to purple towards apex, apex white | Suffused green towards apex and margins, prominent reddish brown at inner side, minutely spotted at base |
| Synsepalum shape | Narrowly ovate, dorsally convex | Narrowly to broadly ovate, dorsally convex, apex acute | Narrowly to broadly ovate, dorsally convex, apex acute | Cucullate, ovate, apex obtuse, base cordate |
| Synsepalum size | 1.8–3.0 × 1–1.6 cm | 2.2–2.9 × 1.5–2 cm | 2.2–2.9 × 1.5–2 cm | 3.5 × 2.1 cm |
| Synsepalum texture | Sparsely hairy, dorsal mid-vein 2-keeled | Sparsely hairy | Sparsely hairy | Back side outwardly keeled |
| Synsepalum colour | Green | Greenish at base, suffused purple towards margin, apex white | Greenish at base, suffused purple towards margin, apex white | Yellowish green, venation green, bordered white, suffused green, blotched dark brown at base as in dorsal sepal |
| Pouch shape | Apex prominently emarginated, side lobes incurved | Apex prominently emarginated, side lobes incurved | Apex prominently emarginated, side lobes incurved | Side lobes rectangular, incurved |
| Pouch size | 2.8–4 × 1.5–2 | 3.2–4 × 2–2.2 | 3.2–4 × 2–2.2 | 2.8 × 1.4 cm |
| Pouch texture | Glossy to matt, side lobes verruculose, warts irregular shape | Glossy to matt, margin ciliolate, indumentum purple, verruculose, warts purple to black, variously shaped | Glossy to matt, margin ciliolate, side lobes glossy, verruculose, warts maroon | Glossy, hairy interior at the nectary and dorsal opening |
| Pouch colour | Pinkish to brownish purple | Pale brown, maroon purple to maroon, often suffused greenish on the underside | Reddish brown to maroon | Reddish green, pale green at mouth, venation brown |
| Staminode shape | Somewhat transversely reniform; base bilobed, lobes convex across its width, to 1.5 mm long; apical teeth three, the central tooth lobe-like, sometimes revolute, lateral apical teeth acute | Tranverse lunate; base bilobed, lobes laterally convex, to 3 mm long; apical lateral teeth falcate, sometimes slightly incurved, apical central tooth much reduced, lobe-like, sometimes with marginal lobes | Tranverse lunate; base bilobed, lobes laterally convex, to 3 mm long; apical lateral teeth falcate, usually prominently incurved, apical central tooth much reduced, sometimes with marginal lobes | Obovate |
| Staminode size | 7–8 × 6.5–8 mm | 8–11 × 10–15 mm | 7–8 × 6.5–8 mm | 8 × 8 mm |
| Staminode texture | Glossy, glabrous | Sparsely hairy | Sparsely hairy | Verruculose, hairy, indumentum dark brown |
| Staminode colour | Pale yellow to green with a patch of bright green pattern laterally across each side of its centre | Pale green, pale purple or green suffused purple with a patch of green to purple pattern laterally across each side of its centre | Cream to pale green, often suffused pale purple with a patch of green to purple pattern laterally across each side of its centre | Yellowish green |

**B.**

| Characters | *C. monophylla* [2] | *C. taenioides* [2] | *C. chrysoglossoides* (Besi *et al*. EDW122) |
| --- | --- | --- | --- |
| Pseudobulbs | Small, each bearing a single leaf | Small, each bearing only one full-sized leaf | Clustered, single-leaved |
| Leaves shape | Ovate, apex acute; petiole 7.5–15 cm long | Elliptic, apex acuminate or acute; petiole 10–18 cm long | Ovate to wide elliptic, apex acuminate, abruptly narrowed to a ca. 10–15 cm long and sulcate petiole, plicate |
| Leaves size | 19 × 7.5 cm | 15–20 × 6–7.5 cm | ca. 20 × 8 cm |
| Leaves texture | Pubescent along the nerves and beneath | Glabrous above, finely pubescent below | Entire but very shortly pubescent beneath |
| Inflorescences | Erect, shortly pubescent; peduncle up to 30 cm long; rachis much shorter, 4- to 5-flowered | Densely pubescent; peduncle ca. 20 cm long; rachis ca. 8 cm long, 10- to 15-flowered, velvet pubescent | ca. 30–40 cm long, arised from pseudobulbs which just forming, emerging together with the leaf |
| Floral bracts | Persistent, lanceolate, apex acuminate, 5 mm long | Persistent | Narrowly triangular, very acute, 5 mm long, greenish yellow, persistent |
| Flowers | Pinkish-white, nodding, not widely opening, well-spaced | Yellowish-white, labellum callus yellowish, sepals pubescent at dorsal surface | Pinkish-white, opening widely, glabrous inside, finely pubescent outside; labellum pale pink with a central orange mark |
| Dorsal sepal shape | Ovate with slender apices, shortly pubescent | Linear, tapering from the base, apex acute | Oblong-ovate, apex acuminate |
| Dorsal sepal sizes | 1.3 × 0.5 cm | 1.2 × 0.2 cm | 1.2 × 0.5 cm |
| Lateral sepals | More or less similar as dorsal sepal | Obliquely linear | 1.2 × 0.4 cm, obliquely elliptic, apex acuminate |
| Petals | 0.8 × 0.3 cm | 1.15 × 0.12 cm, thin-textured, linear, sparsely pubescent at dorsal surface | 1 × 0.3 cm, oblong-ovate, apex acuminate, base cuneate, finely pubescent outside |
| Labellum shape and size | 3-lobed; side lobes near base, short, rounded; mid lobe clawed, apically bilobed, 4 mm broad apically, narrower basally, the lobules diverging and rounded; callus with 2 keels on the claw of the midlobe | 3-lobed; side lobes small, semi erect, obliquely triangular; mid lobe clawed, longer than side lobes, oblong, long-acuminate; callus with 3 keels extending from the base to the middle | 3-lobed; side lobes very small and rounded, 1 mm long; mid lobe clawed, spade-shaped, almost circular, disk slightly concave, minutely apiculate, base cuneate, a deep median ridge running from the base to apex, a 5 mm long linear and verrucose callus at base |

**C.**

| Characters | *L. zollingeri* [3] | *L. brachystachys* (Besi *et al*. HS113) |
| --- | --- | --- |
| Stems | Simple or branched stems, internodes 1–2.5 cm long. | Suberect or curved, branching, internodes 1–2 cm |
| Leaves shape | Terete, apex obtuse | Terete, apex obtuse |
| Leaves size | 12–17 × 0.2–0.4 cm | 5–13 × 0.2–0.3 cm |
| Racemes | 0.9–1.5 cm long, 3 to 9-flowered; peduncle 3 mm long | 2 cm long, 2- to 4-flowered; rachis 1 cm; peduncle 8 mm long |
| Flowers | Petals about as long as the dorsal sepal, but wider with which they are joined together forming a hood, greenish | Petals distinctly narrower than the dorsal sepal |
| Dorsal sepal shape | Ovate, apex obtuse to rounded | Ovate, apex obtuse |
| Dorsal sepal size | 4–6 × 3–4 mm | 4 × 2 mm |
| Lateral sepals shape | 4.8–7 × 1–2 mm, keel prominent up to 0.2 mm wide, apex about squarely cut off | 5 × 2.5 mm , ovate-oblong, slightly hooded, dorsally slightly carinate, keeled abaxially, keel becoming winged at apex, apex obtuse |
| Petals shape | 4.8–6 × 3–4 mm, ovate, somewhat oblique, apex obtuse to rounded | 5 × 1 mm, linear-oblong, apex obtuse |
| Labellum shape | Bilobed, distinct incurved and erect two lobes at base | Simple, more or less rectangular, indistinct lateral lobes at base |
| Labellum epichilium | 2.5–3 × 3.5–5 mm, heart-shaped to transversal rectangular, concave, apex rounded to obtuse or acute to more or less obtuse | ca. 2 × 3 mm, suborbicular or heart-shaped, apex obtuse |
| Labellum hypochilium | More or less rectangular to quadrangular, concave,  two-lobed, surface in the middle with a longitudinal groove | Quadrangular, thick, concave, grooved at the middle |
| Labellum size | 5–6 × 3–5 mm | 5 × 3 mm |
| Labellum texture | Glabrous, hypochilium separated from the epichilium by a shallow vague or a distinctly groove | Glabrous, fleshy, deep indentation between epichilium and hypochilium |
| Gymnostemium | 3 × 1–2 mm; stigma large, more or less orbicular | 3 × 2 mm; stigma large, orbicular |
| Anther-cap | 1.5–2 mm | 1 × 2 mm |

**D.**

| Characters | *H. paradiseoides* [4] | *H. rostellifera* (Besi *et al*. EDW108) |
| --- | --- | --- |
| Stems | Short | ca. 20 cm tall |
| Leaves shape | Linear, apex acute | Narrowly oblanceolate-oblong, apex acute, often apiculate |
| Leaves size | 10 × 0.5 cm | 12–15 × 1–1.4 cm |
| Raceme | rachis to 10 cm long | rachis 3 cm long |
| Floral bracts | Triangular acuminate, to 1.5 cm long | Lanceolate, apex acuminate, 0.8 cm long |
| Flowers colour | White | Pale salmon-pink |
| Dorsal sepal shape | Broadly elliptic | Suborbicular, apex cuspidate |
| Lateral sepals shape | 5.5 mm long, oblong, oblique, recurved, in-rolled | 6 mm, ovate to rounded, obliquely, reflexed |
| Petals shape | Obliquely lanceolate | Oblong, apex cuspidate |
| Labellum midlobe | Linear, apex obtuse, 7 mm long | Linear to oblong, apex rounded, 11 mm long |
| Spur | 1.4 cm long, thickened towards the base, curved forward | ca. 1.7 cm long, geniculate, thickened, slightly folded apically |
| Column | 2.0 mm long by 2.5 mm wide; anther canals compact, short, bent upwards | 2.1 mm long; anther canals ca. 5 mm long, geniculate at the middle |

**E.**

| Characters | *B. pudicum* [5] | *B. cordiferum* subsp. *borneense* |
| --- | --- | --- |
| Pseudobulbs | 1–2 cm long, conical | 2–3 cm long, ovate-lanceolate |
| Leaves shape | Oblanceolate, apex acute, conduplicate, one per pseudobulb | Linear-lanceolate, apex attenuate and unequally bilobulate, conduplicate towards base |
| Leaves sizes | 18.5–19.5 × 1.9 × 2 cm | 13–20 × 1.1–1.8 cm |
| Racemes | Subterminal or axillary, ascending, tomentose white, ca. 5.5 cm long; peduncle 2.5 cm long; rachis 3 cm long | Axillary, covered by short white indumentum; peduncle 1.8 cm long; rachis 3.5 cm long |
| Floral bracts | Lanceolate, green, apex acute and purplish, ca. 2 mm long | Ovate, subacute, minutely puberulus, 3.2 mm long |
| Flowers | Scented, dorsal surface pubescent, whitish with apex of the sepals tinged purple | Unscented, pubescent dorsally, reddish, veins darker, petals and lateral sepals reflexed |
| Dorsal sepal | 3 × 1.5 mm, ovate, apex obtuse, concave | 5.3 × 2 mm, oblong, obtuse, concave |
| Lateral sepals | 3.5 mm × 2 mm, broader than dorsal sepal | 4.4 × 3.5 mm, obliquely ovate, falcate, obtuse, cucullate |
| Petals | 2 mm × 1 mm, oval, apex obtuse | 4.6 × 1.2 mm, linear-oblong, obtuse, falcate, glabrous |
| Labellum | 3-lobed, 2.5 mm long | 3-lobed, 4.5 mm long |
| Labellum side lobes | 0.5 mm long, erect, triangular | ca. 0.9 mm long, erect, widely rounded |
| Labellum midlobe | 1 × 2 mm, decurved, base triangular | Triangular-ovate, acute, fleshy, with widely ovate callus, ca. 1.2 mm long; disc with a median keel, 2 parallel and oblong fleshy lamellae extending from near the base to the base of midlobe, ca. 1.5 mm long; base cuneate |
| Mentum | 2 mm long | 3 mm long |
| Column | 1 mm long | 4.5 mm long |

**F.**

| Characters | *T. campanulatum* [6] | *T. stella* [6] | *T. intermedium* [6] | *T. rugulosum* (Besi *et al*. HS107) |
| --- | --- | --- | --- | --- |
| Stem | Short | Stout, densely rooting, up to ca. 6 mm long | Rather stout, sometimes branched, densely rooting,  up to ca. 7 mm long | 1–3 mm long |
| Roots | Elongate, fleshy, appressed to the substratum, flattened above and beneath, grey-green, 0.7 to  1.5 mm diameter | Elongate, appressed to the substratum, strongly flattened above and beneath, thin, ribbon-like, rather shiny dark green above, much paler beneath, ca. 1.3–3 mm wide,  ca. 0.5 mm thick | Elongate, appressed to the  substratum, flat, taeniaeform, very slightly rounded above and rather shiny dark green, up to ca. 2mm wide, ca. 0.5  mm thick | Thin, flattened, wrinkled, 3–15 × 0.1–0.3 cm, green |
| Inflorescences | Erect or erecto-patent, many-flowered, peduncle filiform thickened upwards 3–6 mm long, rachis flexuous sinuous ca. 3 mm long | Erect, many-flowered;  peduncle terete, thickened upwards with 1 or 2 small  sheaths at the base and another some distance above, very shortly muriculate, dark green, 4-8 mm long; rachis  erect or flexuous, sinuous, sides minutely muriculate,  flattened or grooved inside, dark green, 3–5 mm long, internodes ca. 1 mm long | Erect or erecto-patent, few-flowered; peduncle filiform, slightly thickened upwards with 1 or 2 small sheaths at the base and ca. 2 at intervals above, very shortly muricate, dark green, 1–2.25 cm long; rachis as thick as apex of peduncle, erect or flexuous, sinuous, very shortly muricate, dark green, up to ca. 7.5 mm long,  internodes 1 to 1.7 mm long | An axillary raceme, ca. 1–1.5 cm long, peduncle filiform, glabrous, apex with alternate bracts, green |
| Floral bracts | Alternate, triangular-ovate, acute | Alternate, appressed to the pedicel, ovate-triangular, acutely apiculate, inside concave, outside convex minutely muriculate, dark green, ca. 0.7 mm long and as broad | Alternate, base appressed to the pedicel, roundly triangular, acutely apiculate,  inside concave, outside convex very shortly muriculate, dark green, ca. 0.7 mm long and as broad | Alternate, green, less than 1 mm long |
| Flowers | Appearing 1-2 together at short intervals and lasting more than 1 day, campanulate, whitish, 3.5-4.2 mm long. Sepals and petals adnate to well beyond the middle into a tube, 1-nerved, outside subverruculose, in texture rather fleshy | Appearing singly or in  pairs at intervals, opening in the evening and lasting one  night, 0.90-1.25 cm. long to end of spur, 0.80-1 cm. wide, pale yellowish white to bright yellow or salmon pink. Sepals and petals 1-nerved, adnate at base in the form of a tube for ca. 1.7 mm. | Appearing singly or in pairs at intervals opening in the  evening and lasting 1 night, well expanded, pale salmon  pink, ca. 0.50 cm. long. Sepals and petals 1-nerved, shortly adnate at the base into a tube, free part recurved spreading subulate obtuse | 1 or 2 per inflorescence, resupinate, porrect, tubular, 5 × 2 mm, yellowish green, borne successively, spur 1 mm long, tubular; buds, open flowers and capsules often present simultaneously; sepals, petals and labellum almost having the same length, barely distinguishable. Sepals and petals somewhat fleshy, basally fused, spreading only in upper half, triangular |
| Sepals | Adnate to the lip, in all 2.5-3 mm long, free parts  hardly expanded almost parallel; free part of sepals  triangular, obtuse, ca. 1 mm long ca. 0.9 mm wide | Free part of dorsal sepal subulate, acute, ca. 5.5 mm long, ca. 1 mm wide. Lateral sepals adnate to the lip at the entrance to the spur, free part subulate acute or subacute ca. 5 mm long ca. 1.5 mm wide | Free part of dorsal sepal ca. 3 mm long, ca. 0.5 mm wide. Free part of lateral sepals ca. 3 mm long,  ca. 0.6 mm wide. | Dorsal sepal free part ca. 3.3 × 0.9 mm, narrowly triangular, apex acute. Lateral sepals free part ca. 3.2 × 0.5 mm, triangular, wider at base, apex acute, longer than petals |
| Petals | Free part of petals triangular-ovate, obtuse, ca. 0.9 mm long and as wide | Free part of petals linear-lanceolate, acute, ca. 5 mm long, ca. 0.9 mm wide | Free part of petals ca. 2.8 mm long, ca. 0.5 mm wide. | Petals free part ca. 3 × 0.5 mm, narrowly triangular, apex acute |
| Labellum | 3-lobed spurred, spread out without the spur ca. 2.4-2.8 mm long, ca. 1.1-1.3 mm wide across the side lobes | Entire, spurred, blade rhombeolanceolate,  acuminate, acute, concave inside | Fleshy, entire, spurred; blade subulate, subacute,  concave, produced below the apex to an incurved seta  ca. 0.8 mm long | ca. 3 × 0.7 mm, unlobed, pyriform when flattened; apex small, obtuse, upcurved, falcate; spur 1 mm long |
| Column | Very short, green, ca. 0.5 mm long, stigmatic arms oblong obtuse | Short, straight, green, stigmatic arms parallel oblong obtuse projecting down into the excavation  at the base of the lip | Short, dilate upwards, papillose towards the apex,  back produced at apex to a large ovate obtuse fleshy lobe, stigmatic arms long parallel linear-oblong obtuse projecting down into the excavation at the base of the lip, green | Porrect, at slight angle to ovary, less than 1 mm long |

**References**

1. Leong PKF. Flora of Peninsular Malaysia – Cypripedioideae. Malesian Orchid J. 2014; 13: 113-127.
2. Clayton D, Cribb P. The Genus *Calanthe*. Malaysia, Kota Kinabalu: Natural History Publications; 2013.
3. Sulistiarini D. The orchid genus *Luisia* in Indonesia. Reinwardtia 2014; 10(4): 383-398.
4. Jutta M, Faridah QZ. *Malaxis inexpectata* and *Habenaria paradiseoides* (Orchidaceae), new records for Peninsular Malaysia. Gard Bull (Singapore) 2005; 57: 263-267.
5. Tang CH. *Revision of the genus Eria (Orchidaceae)* [Master Dissertation]. Malaysia: University Putra Malaysia; 2007. pp. 29-30.
6. Carr CE. The Genus *Taeniophyllum* in the Malay Peninsula. Gard. Bull. Straits Settlem. 1932; 7: 61-82.
