# Supplementary material for: Rare orchid species in Malaysia: New records, recollections and amended descriptions
Source: PLoS One. 2022 Apr 26;17(4):e0267485. doi: 10.1371/journal.pone.0267485 (PMC9041863; doi:10.1371/journal.pone.0267485)
Supplement: S2 File — A. Paphiopedilum exul (Ridl.) Rolfe. B. Calanthe chrysoglossoides J.J.Sm.. C. Luisia brachystachys (Lindl.) Blume. D. Habenaria rostellifera Rchb.f.. E. Bryobium cordiferum subsp. borneense (J.J.Wood) Schuit. F. Taeniophyllum rugulosum Carr. (DOCX) [file pone.0267485.s002.docx]

**S2 File. List of specimens examined, including information on localities, collectors, date of collection, and habitat.** A. *Paphiopedilum exul* (Ridl.) Rolfe. B. *Calanthe chrysoglossoides* J.J.Sm.. C. *Luisia brachystachys* (Lindl.) Blume. D. *Habenaria rostellifera* Rchb.f.. E. *Bryobium cordiferum* subsp. *borneense* (J.J.Wood) Schuit.. F. *Taeniophyllum rugulosum* Carr.

**A.**

| No | Specimen ID/Barcode | Type material | Herbarium | Country | Locality | Date | Collector | Habitat |
| --- | --- | --- | --- | --- | --- | --- | --- | --- |
| 1 | 0141768 | Extra | SING | Thailand | Peninsular Thailand, Kasoom, Phangnga, | Nov 1897 | Curtis, C | On rock |
| 2 | 0141766 | Extra | SING | Thailand | Peninsular Thailand | 1892 | Ridley, HN | N/A |
| 3 | 0056352 | Holotype  **Type Taxon:** *Cypripedium insigne* var. *exul* | SING | Thailand | Central Thailand, Bangkok | 1891 | Ridley, HN | N/A |
| 4 | K000595625 | Extra | K | Thailand | Central Thailand, Bangkok | 2 April 1925 | Kerr, AFG | N/A |
| 5 | 0164242 | Extra | SING | N/A | N/A | 28 January 1921 | Deshmukh, GB | N/A |
| 6 | 0141767 | Extra | SING | Thailand | Peninsular Thailand | 12 December 1918 | Mhd Haniff | N/A |
| 7 | EDW113 | Extra | UPM | Malaysia | Perlis | 31 October 2019 | Besi *et al*. | Limestone forest |

**B.**

| No | Specimen ID/Barcode | Type material | Herbarium | Country | Locality | Date | Collector | Habitat |
| --- | --- | --- | --- | --- | --- | --- | --- | --- |
| 1 | 1506, 1165 | Syntype | BO | Indonesia | Java, Gunung Salabintana (Selabintana) | N/A | Jackson | Growing in quite rich soil in heavy shade |
| 2 | L.1495231 | Extra | NHN | Indonesia | Sumatra, East Coast, Aek, Oessim Asahan | 3 October 1936 | Rahmat Si Boeea | N/A |
| 3 | L.1495232 | Extra | NHN | Indonesia | Sumatra, East Coast, Aek, Oessim Asahan | 12 October 1936 | Rahmat Si Boeea | N/A |
| 4 | L.1495233 | Extra | NHN | Indonesia | Sumatra, Toba, residency of Tapianoeli, Headwaters of Aek Mandosi | 29 September 1936 | Rahmat Si Boeea | N/A |
| 5 | L.1495234 | Extra | NHN | Indonesia | Sumatra, east coast, Headwaters of Aek Liang, Asahan | 15 October 1936 | Rahmat Si Boeea | N/A |
| 6 | L.1495235 | Extra | NHN | Indonesia | Sumatra, Toba, Boundary between East Coast and Tapianoeli, Vicinity of Taloen na Oeli | 11 November 1936 | Rahmat Si Boeea | N/A |
| 7 | L.1495236 | Extra | NHN | Indonesia | Sumatra, Toba, residency of Tapianoeli, Aek Riman | 13 November 1936 | Rahmat Si Boeea | N/A |
| 8 | L.1495238 | Extra | NHN | Indonesia | Sumatra, east coast, Aek Boeloe Bolon | 7 November 1936 | Rahmat Si Boeea | N/A |
| 9 | L.1495239 | Extra | NHN | Indonesia | Sumatra, east coast, Aek Oessim, Asahan | 3 October 1936 | Rahmat Si Boeea | N/A |
| 10 | L.1495240 | Extra | NHN | Indonesia | Sumatra, east coast, Aek Oessim, Asahan | 3 October 1936 | Rahmat Si Boeea | N/A |
| 11 | L.1495194 | Extra | NHN | Indonesia | Sumatra, Toba, Boundary between East Coast and Tapianoeli, Vicinity of Taloen na Oeli | 1 December 1936 | Rahmat Si Boeea | N/A |
| 12 | L.1495196 | Extra | NHN | Indonesia | Sumatra, east coast, Headwaters of Aek Liang, Asahan | 15 October 1936 | Rahmat Si Boeea | N/A |
| 13 | L.1495197 | Extra | NHN | Indonesia | Sumatra, east coast, Headwaters of Aek Liang, Asahan | 15 October 1936 | Rahmat Si Boeea | N/A |
| 14 | L.1495195 | Extra | NHN | Indonesia | Sumatra, Toba, Residency of Tapianoeli, Vicinity of Loemban Loboe | 27 July 1936 | Rahmat Si Boeea | N/A |
| 15 | L.1495201 | Extra | NHN | Indonesia | Lesser Sunda Islands, Lombok, Rindjani Vulkangebirge | 19 June 1909 | Elbert, J | N/A |
| 16 | L.1495198 | Extra | NHN | Indonesia | North Sumatra, Gunung Leuser Nature Reserves, Atjeh. Upper Mamas River Valley expedition, ca.15km. W. of Kutacane. Camp Pilar to Pawang | 22 June 1979 | Wilde WJJO de; Wilde-Duyfjes BEE de | Mossy forest, 1600 m |
| 17 | L.1495237 | Extra | NHN | Indonesia | Gunung Leuser Nature Reserves, Atjeh, North Sumatra. Upper Mamas River Valley expedition, ca.15km. W. of Kutacane. Camp Pilar to Pawang | 22 June 1979 | Wilde WJJO de; Wilde-Duyfjes BEE de | Moist riverine forest, 1250 m |
| 18 | EDW122 | Extra | UPM | Malaysia | Selangor, Batang Kali | 12 October 2020 | Besi *et al*. | Montane forest, 1000 m |

**C.**

| No | Specimen ID/Barcode | Type material | Herbarium | Country | Locality | Date | Collector | Habitat |
| --- | --- | --- | --- | --- | --- | --- | --- | --- |
| 1 | K000891542 | Syntype,  E.I.C.; Herbarium Hookerianum 1867; sketch given.  “Based on *Mesoclastes brachystachys* Lindl.” Glast.Sp. Arch. p.45. | K | Bangladesh | N/A | N/A | Wallich, N | N/A |
| 2 | K000891534 | Unknown type,  Photo and protologue of *Luisia siamensis* Rolfe ex Downie  attached | K | Thailand | Me Sue, near Chiangmai | 7 April 1912 | Kerr, AFG | On small trees in marsh savannah, 457.0 m |
| 3 | BM000538799 | Extra,  *Luisia indivisa*King & Pantl. | NHM | India | Sikkim | March 1897 | Anderson | N/A |
| 4 | BM000538804 | Extra,  *Luisia indivisa*King & Pantl. | NHM | Myanmar | Tanintharyi, Dawei | May 1897 | H.G. Batten | N/A |
| 5 | BM000538800 | Extra | NHM | India | N/A | April 1899 | N/A | N/A |
| 6 | BM000538802 | Extra | NHM | N/A | N/A | 21 May 1869 | Charles Baron Clarke | N/A |
| 7 | BM000538801 | Extra | NHM | N/A | N/A | 21 April 1872 | Charles Baron Clarke | N/A |
| 8 | K000873796  With K000873795 | Extra | K | N/A | N/A | N/A | Lobb, T | N/A |
| 9 | K000891535 | Extra | K | Thailand | Me Sue, near Chiangmai | 7 April 1912 | Kerr, AFG | On small trees in marsh savannah, 457.0 m |
| 10 | K000891541 | Extra | K | India | Dooars, Mount Anduson | March 1897 | N/A | N/A |
| 11 | K000873795  Illustrations given; With K000873796 | Extra | K | N/A | N/A | 1994 | De Silva, F | N/A |
| 12 | P00324094 | Extra | MNHN  Museum national d'Histoire naturelle | Vietnam | Annam: Nhatrang | 22 March 1960 | Sigaldi, de | N/A |
| 13 | P00324095 | Extra | MNHN  Museum national d'Histoire naturelle | Laos | Brikhane, Wiengchan (Vientiane) | 27 March 1932 | Kerr, AFG | On tree, 200 m |
| 14 | P00324093 | Extra | MNHN  Museum national d'Histoire naturelle | India | Jaintea Hills Assam | April 1899 | Prain, D | N/A |
| 15 | P00324092 | Extra | MNHN  Museum national d'Histoire naturelle | India | Sikkim, Dooars | March 1897 | N/A | N/A |
| 16 | L.1521919 | Extra | NHN | India | Assam. Mishing. Jaintea Hills, Assam. | 1 April 1899, 30 April 1899 | Prain's Collector | N/A |
| 17 | HS113 | Extra | UPM | Malaysia | Bukit Pedu, Kedah | 12 August 2020 | Best *et al*. | Conglomerate hilss |

**D.**

| No | Specimen ID/Barcode | Type material | Herbarium | Country | Locality | Date | Collector | Habitat |
| --- | --- | --- | --- | --- | --- | --- | --- | --- |
| 1 | 0047370 | Holotype | SING | Thailand | Peninsular Thailand, Trang | 30 May 1919 | Mhd Haniff | 914 m a.s.l. |
| 2 | 0180012 | Holotype of *Habenaria roseata* Ridl. | SING | Thailand | Peninsular Thailand, Trang, Cultivated in the Penang Gardens | 29 June 1906 | N/A | N/A |
| 3 | 0180011 | Unknown type | SING | Thailand | Peninsular Thailand, Trang, Kantang | 30 May 1919 | Mohd Haniff; Mohd Nur, #SFN4300 | N/A |
| 4 | 0139971 | Extra | SING | Malaysia | Peninsular Malaysia, Terengganu, Rantau Abang | 1903 | Down, VB St. | N/A |
| 5 | 0139970 | Extra | SING | Malaysia | Peninsular Malaysia, Kedah, Baling | 13 August 1940 | Nauen, JC | N/A |
| 6 | 0139969 | N/A | SING | Malaysia | Peninsular Malaysia, Kedah, Kpg. Naka | 9 September 1933 | Holttum, RE | 30 m a.s.l. |
| 7 | NY00008930 | Unknown type | The William and Lynda Steere Herbarium of the New York Botanical Garden (NYBG) | N/A | N/A | N/A | N/A | N/A |
| 8 | K000595796 | Extra | K | Thailand | Chiangmai | 27 September 1921 | Kerr, AFG | N/A |
| 9 | K000595797 | Extra | K | Thailand | Banang Sta, Pattani | 25 July 1923 | Kerr, AFG | Savannah |
| 10 | K000595798 | Extra | K | Thailand | Banang Sta, Pattani | 25 July 1923 | Kerr, AFG | Savannah |
| 11 | K000595793 | Extra | K | Thailand | Sadao, Songkala | 22 July 1928 | Kerr, AFG | Open grassy forest, 50 m |
| 12 | K000595794 | Extra | K | Thailand | Kanchanadit, Surat | 01 August 1927 | Kerr, AFG | Common in savannah, 5 m |
| 13 | K000595791 | Extra | K | Thailand | Doi Sutep | 29 August 1909 | Kerr, AFG | In (illegible) jungle or edge of paddy ground, 305.0-549.0 m |
| 14 | K000595799 | Extra | K | Thailand | Banang Sta, Pattani | 25 July 1923 | Kerr, AFG | Common in savannah |
| 15 | K000595790 | Extra | K | Thailand | Doi Sutep | 29 August 1909 | Kerr, AFG | 305.0-549.0 m |
| 16 | K000595800 | Extra | K | Thailand | Banang Sta, Pattani | 25 July 1923 | Kerr, AFG | Common in savannah |
| 17 | K000827011 | Extra | K | China | Mentze, Yunnan | June 1893 | Hancock, W | Damp grassy slopes, 1676.0-1829.0 m |
| 18 | K000595795 | Extra | K | Thailand | Kanchanadit, Surat | 01 August 1927 | Kerr, AFG | Common in savannah, 5 m |
| 19 | K000595792 | Extra | K | Thailand | Doi Sutep | 11 November 1914 | Kerr, AFG | On paddy feild (illegible) at the foot of Doi Sutep, 300.0 m |
| 20 | K000364319 | Extra, Com 8/74; description given; Seen for the Flora of Thailand, H. Kurzweil, 03 October 2007 | K | Thailand | N/A | 17 August 1984 | Godefroy-Lebeuf, A | N/A |
| 21 | L.1516483 | Extra | NHN | Thailand | Chiang Mai, District: Chiang Dao, Ban Jong village area, Wieng Haeng Subdistrict. | 16 September 1989 | Maxwell, JF | Open grassy, fire prone area in deciduous dipterocarp-oak, pine forest, shale bedrock, 975 m |
| 22 | L.0770530 | Extra | NHN | Thailand | Chiang Mai, District: Muang, Doi Sutep-Pui National Park, east side, Gukao Falls area. | 4 October 1989 | Maxwell, JF | N/A |
| 23 | L.1516482 | Extra | NHN | Thailand | Chiang Mai, District: Muang, Doi Sutep-Pui National Park, east side, Gukao Falls area. | 4 October 1989 | Maxwell, JF | Open moist seepage area in thin soil on a granite outcrop in the deciduous dipterocarp-oak forest, granite bedrock, fire prone area |
| 24 | L.1516481 | Extra | NHN | Thailand | South-Eastern; Thailand, S.E.: Chanburi, Makham. | 3 June 1965 | Phengklai, C | Open space |
| 25 | L.1516480 | Extra | NHN | Thailand | Peninsular Thailand,Tung Song | 19 July 1929 | Rabil, NB | N/A |
| 26 | L. 0597707 | Extra | NHN | Thailand | Chiang Mai, District: Chiang Dao, Ban Jong village area, Wieng Haeng Subdistrict. | 16 September 1989 | Maxwell, JF | N/A |
| 27 | L.1516479 | Extra, *Habenaria roseata*  Ridl. | NHN | Thailand | Thailand, Surat, 14 km north of Chumphon | 11 August 1966 | Larsen, K; Smitinand, T; Warncke, E | N/A |
| 28 | BM000034956 | Extra | NHM | China | N/A | N/A | B.M.Bartholomew & D.E.Boufford | N/A |
| 29 | BM000033437 | Extra | NHM | Thailand | Prachuap Khiri Khan | 8 November 1931 | Alexander Marcan | N/A |
| 30 | 00140709 | Extra | AMES | China | Yunnan, Kunming | 17 August 1984 | B. Bartholomew & D. E. Boufford | Grounds of the Kunming Institute of Botany, Heilongtan, Kunming Municipality, 2000 m |
| 31 | 00140671 | Extra | AMES | China | Yunnan, Mts of the Yangpi River drainage basin. Along Yangpi road | N/A | J. F. Rock | N/A |
| 32 | 00140670 | Extra | AMES | China | Yunnan, Simao, Tze-moa | N/A | Henry, A | N/A |
| 33 | 00140669 | Extra | AMES | China | Yunnan, Mengzi | N/A | Henry, A | N/A |
| 34 | 00140672 | Extra | AMES | China | Yunnan | N/A | Maire, EE | N/A |
| 35 | 01946515 | Extra | AMES | China | N/A | N/A | Teng, SW | N/A |
| 36 | 01946805 | Extra | AMES | Thailand | Songkla. Haad Yai. Klong Hoy Kong. | 21 November 1984 | Maxwell, JF | Open savannah, moist soil. |
| 37 | P00370734 | Extra | MNHN | Thailand | Northeast Thailand, Loie, Sitan | 16 August 1948 | Royal Forest Department | Ground orchid, common in mixed forest, 300 m |
| 38 | P00439735 | Extra | MNHN | Vietnam | Rüng Bbon Phu | August 1967 | Dournes, J | N/A |
| 39 | EDW108 | Extra | UPM | Malaysia | Perlis | 2 August 2020 | Besi *et al*. | N/A |

**E.**

| No | Specimen ID/Barcode | Type material | Herbarium | Country | Locality | Date | Collector | Habitat |
| --- | --- | --- | --- | --- | --- | --- | --- | --- |
| 1 | 42172 | Isotype | K | Malaysia | Sarawak, Gunung Mulu National Park | 14 March 1978 | Nielsen, I | 400 m a.s.l. |
| 2 | EDW068 | Extra | UPM | Malaysia | Kapit | 2 December 2019 | Besi *et al*. | Riparian forest, 50 m a.s.l. |

**F.**

| No | Specimen ID/Barcode | Type material | Herbarium | Country | Locality | Date | Collector | Habitat |
| --- | --- | --- | --- | --- | --- | --- | --- | --- |
| 1 | 0162774 | Extra | SING | Malaysia | Peninsular Malaysia, Pahang, Sungai Sat, Ulu Tembeling | 1929-07-22 | Henderson, MR | On small riverside trees. |
| 2 | K000942486 | Type | K | Malaysia | Peninsular Malaysia, Pahang, Sungai Sat, Ulu Tembeling | August 1929 | Carr, CE | Riparian, on Saraca tree, 152 m |
| 3 | HS107 | Extra | UPM | Malaysia | Perak, Lata Kekabu | 3 April 2021 | Besi *et al*. | Riparian |
